# A scoping review of applications of the Consolidated Framework for Implementation Research (CFIR) to telehealth service implementation initiatives

**DOI:** 10.1186/s12913-022-08871-w

**Published:** 2022-11-30

**Authors:** Pavani Rangachari, Swapandeep S. Mushiana, Krista Herbert

**Affiliations:** 1 Department of Population Health and Leadership, School of Health Sciences, University of New Haven, 300 Boston Post Road, West Haven, CT 06516 USA; 2 Veterans Affairs (VA) Quality Scholars Program - San Francisco VA Healthcare System, San Francisco, CA 94121 USA; 3 Portland Veterans Affairs (VA) Healthcare System, Portland, OR 97239 USA

**Keywords:** Telehealth, Telemedicine, Consolidated framework for implementation research, Implementation barriers or facilitators, Implementation-effectiveness

## Abstract

**Background:**

The Consolidated Framework for Implementation Research (CFIR), introduced in 2009, has the potential to provide a comprehensive understanding of the determinants of implementation-effectiveness of health service innovations. Although the CFIR has been increasingly used in recent years to examine factors influencing telehealth implementation, no comprehensive reviews currently exist on the *scope of knowledge gained exclusively from applications of the CFIR to telehealth implementation initiatives*. This review sought to address this gap.

**Methods:**

PRISMA-ScR criteria were used to inform a scoping review of the literature. Five academic databases (PUBMED, PROQUEST, SCIDIRECT, CINAHL, and WoS) were searched for eligible sources of evidence from 01.01.2010 through 12.31.2021. The initial search yielded a total of 18,388 records, of which, 64 peer-reviewed articles met the inclusion criteria for the review. Included articles were reviewed in full to extract data, and data collected were synthesized to address the review questions.

**Results:**

Most included articles were published during or after 2020 (64%), and a majority (77%) were qualitative or mixed-method studies seeking to understand barriers or facilitators to telehealth implementation using the CFIR. There were few comparative- or implementation-effectiveness studies containing outcome measures (5%). The database search however, revealed a growing number of protocols for implementation-effectiveness studies published since 2020. Most articles (91%) reported the CFIR Inner Setting domain (e.g., leadership engagement) to have a predominant influence over telehealth implementation success. By comparison, few articles (14%) reported the CFIR Outer Setting domain (e.g., telehealth policies) to have notable influence. While more (63%) telehealth initiatives were focused on specialty (vs primary) care, a vast majority (78%) were focused on clinical practice over medical education, healthcare administration, or population health.

**Conclusions:**

Organized provider groups have historically paid considerable attention to advocating for telehealth policy (Outer Setting) reform. However, results suggest that for effective telehealth implementation, provider groups need to refocus their efforts on educating individual providers on the complex inter-relationships between Inner Setting constructs and telehealth implementation-effectiveness. On a separate note, the growth in implementation-effectiveness study protocols since 2020, suggests that additional outcome measures may soon be available, to provide a more nuanced understanding of the determinants of effective telehealth implementation based on the CFIR domains and constructs.

**Supplementary Information:**

The online version contains supplementary material available at 10.1186/s12913-022-08871-w.

## Introduction

Telehealth is known to help patients overcome two barriers they face when seeking health care: distance and time [1, 2]. Proponents of telehealth have argued that it has the potential to transform healthcare delivery by reducing costs, increasing quality of care, enhancing patient & provider satisfaction, and improving population health outcomes [1–5]. Throughout the COVID-19 pandemic, the healthcare industry has witnessed a massive acceleration in the use of telehealth services, bolstered largely by the temporary removal of policy-level barriers to telehealth use (e.g., federal or state restrictions to telehealth coverage and reimbursement) [6]. Although uncertainties remain regarding the future sustainability of telehealth, a key point of consensus, is that the permanent removal of policy-level barriers by itself, would not be sufficient to ensure widespread, sustainable use of telehealth services in the post-pandemic era (although it could serve as an important facilitator) [1, 2, 6]. On the other hand, the literature has emphasized the need for healthcare providers and organizations to make systematic and concerted efforts towards *effective implementation* of telehealth services, for long-term sustainable use [1, 2, 5, 6].

### Existing literature on telehealth service implementation frameworks

Over the past three decades, a variety of frameworks have been put forth for understanding the key barriers and facilitators to implementing telehealth services. Notably, a comprehensive review of ‘telehealth service implementation frameworks,’ by van Dyk (2014) [7] identified nine different frameworks. 1) The seven core principles for the successful implementation of telemedicine, which emphasizes the importance of pragmatism, user-friendliness, user-training, and organizational structure in telehealth implementation [8]; 2) Telehealth readiness assessment tools, which emphasize planning, technological, learning, societal, and policy readiness [9, 10]; 3) Barriers to the diffusion of telemedicine, which emphasizes technical, behavioral, economic, and organizational barriers [11]; 4) Lessons in telehealth service innovation, which identify various success factors, including the policy context, perceived benefit, professional roles and willingness to cross boundaries [12, 13]; 5) The Unified Theory of Acceptance and Use of Technology (UTAUT), which describes the interaction among variables influencing technology acceptance [13]; 6) The Khoja-Durrani-Scott (KDS) Framework, which considers various stages in the telehealth lifecycle [14]; 7) The framework on health system challenges in scaling up for telehealth, which includes consideration for policy, organizational, technological, and financial challenges [15]; 8) The layered telemedicine implementation model, which identifies determinants of success associated with each lifecycle phase of telemedicine [16]; and 9) The comprehensive telemedicine evaluation model, which considers several issues related to telehealth implementation, including the cost of education, quality of clinical services, and community access to services, among others [17]. The review (by van Dyk, 2014) concluded that a “holistic” approach is needed to telehealth service implementation, which includes consideration for organizational structures, change management, technology, economic feasibility, societal impacts, perceptions, user-friendliness, evidence and evaluation, and policy and legislation [7].

It would be relevant to note however, that van Dyk’s (2014) review of telehealth implementation frameworks did not include the Consolidated Framework for Implementation Research (CFIR) [18]. The CFIR was introduced in 2009, to serve as a comprehensive meta-theoretical framework that could be used to inform both ‘implementation science’ and ‘implementation strategy,’ in health services delivery. Since its introduction, the CFIR has been leveraged to inform effective implementation of a variety of health service innovations, including evidence-based practices for patient safety, best practices for patient-and-family centered care, and a variety of health information technologies, including Electronic Health Records and clinical decision support systems [19]. Along these lines, in recent years, the CFIR has also been utilized to inform the implementation of telehealth service initiatives [6].

The CFIR comprises five major domains (characteristics of the intervention, the outer setting, the inner setting, characteristics of the individuals involved, and the process by which implementation is accomplished) [18]. Each domain in turn, is mapped to an array of constructs informed by existing implementation theories and conceptual models. For example, the domain of *inner setting* been mapped to the following constructs: 1) structural characteristics, 2) networks and communication, 3) culture, including norms and values of an organization, and 4) implementation climate or the absorptive capacity for change. Six sub-constructs contribute to a positive implementation climate for an intervention, including readiness for implementation, compatibility, relative priority, organizational incentives & rewards, goals & feedback, and learning climate. Readiness for implementation in turn, includes three sub-constructs, i.e., leadership engagement, available resources, and access to information & knowledge. The five domains (and constructs) in the CFIR, are known to interact in rich and complex ways to influence implementation effectiveness.

As a pragmatic meta-theoretical framework with a comprehensive taxonomy of domains and constructs, the CFIR may be viewed as a “holistic” approach to understanding barriers and facilitators to implementation, compared to any other existing stand-alone framework for telehealth implementation [18, 19]. For example, no other framework mentioned above, is known to give due to consideration to the influence of ‘organizational culture’ or ‘implementation climate’ on implementation effectiveness. By contrast, the CFIR not only emphasizes both these constructs, but it goes a step further in identifying six sub-constructs contributing to a positive implementation climate, including ‘readiness for implementation,’ and three additional sub-constructs contributing to ‘readiness for implementation.’

By virtue of its comprehensive taxonomy (five domains and multiple interrelated constructs for assessing implementation effectiveness), a distinguishing feature of the CFIR is that it can help to understand *why* a particular implementation initiative succeeded or failed. The CFIR has been leveraged as a framework for guiding formative evaluation of implementation efforts. In addition, the CFIR is known to combine well with other established frameworks that could be used to assess implementation scalability and sustainability, e.g., the RE-AIM (Reach, Effectiveness, Adoption, Implementation, and Maintenance) framework [20]. Supplementing the CFIR with the RE-AIM framework in turn, has potential to serve the dual purpose of providing insight into 1) implementation effectiveness and 2) implementation scalability & sustainability.

### Gaps in the literature

It is noteworthy that the CFIR was introduced to the health services sector only a little over a decade ago. Correspondingly, CFIR applications to the telehealth implementation context have only gained momentum over the past 5–10 years. For example, a keyword search on PubMed of (Telehealth OR Telemedicine) AND (Consolidated Framework for Implementation Research) on March 1, 2022, returned a total of 58 articles. By comparison, the broader keyword search on PubMed of (Telehealth OR Telemedicine) AND (Implementation) on the same day, returned a total of 5587 articles, indicating that the body of literature on telehealth and CFIR, accounts for less than 1% of the broader literature on telehealth implementation on PubMed. It is appropriate to compare the results from the two sets of search terms (mentioned above) on any given academic database (like PubMed) at a given point in time. This is because the broader (latter) search serves to capture the general state of *Implementation Research* related to telehealth or telemedicine (on PubMed). The CFIR or the Consolidated Framework for *Implementation Research* is one of many tools that could be used to guide implementation research. As mentioned earlier, since its introduction in 2009, the CFIR has received a lot of attention in implementation science in general and more specifically, in the context of implementing health services innovations like evidence-based practices and Electronic Health Records [19]. The question of interest to this study is the scope of knowledge gained from ‘CFIR applications to telehealth implementation initiatives.’ Therefore, it is appropriate to compare the results from the two sets of search terms mentioned above, to establish a baseline understanding of existing research on this topic and highlight the gap in the literature. Based on the search results, it is not surprising to note that there are no existing reviews of the literature to characterize the scope of knowledge that has been gained exclusively from ‘*applications of the CFIR to telehealth service implementation initiatives*.’ This paper seeks to address this gap.

### Review objective and review questions

This paper undertakes a comprehensive review of the literature to characterize the scope of knowledge that has been gained from *applications of the Consolidated Framework for Implementation Research (CFIR) to telehealth service implementation initiatives*. The review objective is to “identify and synthesize the literature related to applications of CFIR to telehealth service implementation initiatives.” Correspondingly, this scoping review is expected to be directly relevant to healthcare providers and organizations looking to get started with telehealth and/or to design and implement telehealth services for effective and sustainable use. The specific review questions are outlined below.

1. **What have we learned so far from applications of the CFIR to telehealth service implementation initiatives?**
  - What have we learned about the outcomes (success or failure) of telehealth service implementation initiatives?
  - Has the CFIR been combined with other frameworks to enable assessment of both i) effectiveness and ii) scalability or sustainability of telehealth implementation?
  - Which CFIR domains (or constructs) have been identified as most influential in explaining success or failure of telehealth implementation initiatives?
2. **What are the descriptive characteristics of CFIR applications to telehealth service implementation initiatives?**
  - What healthcare domains (e.g., primary care, emergency care, post-acute care, mental health, oral health, etc.) have CFIR applications to telehealth initiatives focused on?
  - What diagnoses or conditions (e.g., diabetes, stroke, cancer, depression, dementia etc.), have CFIR applications to telehealth initiatives focused on?
  - What target populations (e.g., children, adults, seniors, veterans, etc.), have CFIR applications to telehealth initiatives focused on?
  - What technology areas (e.g., synchronous technologies such as interactive audio/video, or asynchronous technologies such as store-and-forward, remote monitoring, mHealth apps), have CFIR applications to telehealth initiatives focused on?
  - What service areas (e.g., clinical practice/healthcare delivery, medical education, population health management, healthcare administration), have CFIR applications to telehealth initiatives focused on?

### Rationale for a scoping review

According to Sucharew and Macaluso (2019) [21], scoping reviews can be useful for answering broad questions, such as “What information has been presented on this topic in the literature?” which in turn is fully consistent with what this review seeks to accomplish. There have been no comprehensive reviews of the literature to-date, to characterize the scope of knowledge that has been gained exclusively from *applications of the CFIR to telehealth service implementation initiatives*. This paper seeks to address this gap, and the review objective (and questions) in turn, are aligned with this purpose. Moreover, a scoping review is intended to provide an overview of the available research evidence without producing a summary answer to a discrete research question. The questions of this review lend themselves to a scoping review approach (versus other types of review) because they are broad in scope, and the review objective is to describe the available evidence on *applications of CFIR to telehealth service implementation initiatives*, as opposed to addressing a discrete research question (e.g., “what is the relationship between implementation climate and implementation success of telehealth implementation initiatives?”)

## Methodology

The review protocol was developed based on guidelines for scoping reviews provided by the Joanna Briggs Institute (JBI) [22]. The PRISMA-ScR criteria (for scoping reviews) were used to frame the review effort [23]. The protocol was not registered. The review protocol is included in Additional file 1 and the completed PRISMA-ScR checklist is included in Additional file 2.

### Information sources

This scoping review sought to identify published original research articles (including quantitative, qualitative, and mixed-method studies) and review articles, to address the review questions. Since the CFIR was officially introduced in 2009, the following five major academic databases were searched for coverage from 01.01.2010 through 12.31.2021: 1) PUBMED, 2) SCIENCE DIRECT (SCIDEIRECT), 3) PROQUEST, 4) CINAHL, and 5) WEB OF SCIENCE (WoS). The article search was conducted in March 2022. The five databases were selected to ensure maximum coverage across medicine and social science domains. Additional searches were conducted on databases relevant to education (ERIC) and engineering domains (IEEE Explore). However, these searches produced negligible results on the topic of interest, and the latter two databases were excluded from information sources for this review.

### Search strategy

The following two sets of search terms were used to search all five databases for the period 01.01.2010 through 12.31.2021: **1)** (Telehealth OR Telemedicine) AND (Consolidated Framework for Implementation Research); and **2)** (Telehealth OR Telemedicine) AND (CFIR). The full electronic search strategy used on PUBMED is included as an example, in Additional file 3. It would be relevant to note that “Telemedicine” is a National Library of Medicine Medical Subject Heading (MeSH) term that includes the synonyms (entry terms) “mobile health,” “mhealth” and “ehealth.” The resulting total number of records from this initial search, for both sets of search terms combined, was 18,388 records (including 73 from PUBMED, 909 from SCIDIRECT, 17,318 from PROQUEST, 32 from CINAHL, and 56 from WoS). These totals included peer-reviewed (scholarly) journal articles, conference papers, working papers, wire feeds, reports, books, trade journals, dissertations, theses, magazines, and other sources. The next section describes the eligibility criteria that were applied to select articles for final inclusion in this scoping review.

### Eligibility criteria

This review considered original research articles (including clinical trials, quantitative, qualitative, and mixed-method studies) and review articles, that were published in peer-reviewed journals, in English language, and pertained to the scope of the review (i.e., ‘*the application of CFIR to telehealth implementation initiatives*’). Since the CFIR was officially introduced only in 2009, papers published between 01.01.10 and 12.31.21, were included for consideration. All forms of telehealth were considered, including telemedicine, digital health, eHealth and mHealth technologies. Research papers considered for inclusion were based on empirical data, including, but not limited to, data collected from clinical trials, surveys, observations, focus groups, and interviews. Among reviews, systematic and scoping reviews were considered for inclusion.

This scoping review excluded: 1) articles that were not published in peer-reviewed journals (e.g., conference papers, working papers, wire feeds, reports, books, trade journals, dissertations, theses, magazines, and other sources); 2) articles that were not pertinent to the review topic (e.g., papers that did not involve use CFIR or telehealth or both). It also excluded 3) articles that were neither original research nor reviews (e.g., study protocols, editorial articles, discussion papers, theoretical reflections, or any other type of article that did not include a methodology section). Additionally, this scoping review excluded 4) articles that did not meet critical appraisal criteria outlined by the Joanna Briggs Institute (JBI) and the Mixed-Method Appraisal Tool (MMAT). While JBI checklists were used for critical appraisal of qualitative studies, reviews, clinical trials, and cross-sectional studies, the MMAT was used for critical appraisal of mixed-method studies. The templates used for article selection (i.e., eligibility and critical appraisal criteria), are included in Additional file 4.

### Process for selecting sources of evidence

Following the search, all identified citations were collated and uploaded into a reference management system (Zotero 5.0) for initial screening. After removal of duplicates, article titles and abstracts were screened for potential inclusion, based on the eligibility criteria for the review. Articles identified for inclusion based on screening of titles & abstracts, were retrieved in full text for assessment based on the eligibility criteria. Reasons for article exclusion at each stage of the process were noted and have been reported in detail in the Results section. All articles that were selected based on eligibility criteria, were subjected to critical appraisal. Only articles that met the critical appraisal criteria were selected for final inclusion in the review. The results of the search are reported in full using a PRISMA flow chart.

### Process for charting data items

All included articles were reviewed to retrieve two categories of data items, Category #1: data items for characterizing the articles (e.g., Article Name, Authors, Publication Year, Article Type); and Category #2: data items for capturing results based on the review questions (RQ1 and RQ2). Both data categories were retrieved from explicit information presented in the reviewed articles and charted in two separate spreadsheet templates included in Additional file 5. Correspondingly, Additional file 5 constitutes the raw dataset for the study. Together, the two data charting spreadsheets incorporated all the fields needed to capture the data items outlined above. No additional assumptions or simplifications needed to be made in the data charting process.

To elaborate, data items relevant to RQ 1 (“*What have we learned so far from applications of the CFIR to telehealth service implementation initiatives?*”) included: 1a. “Does the article include an outcome measure of intervention or implementation effectiveness of the telehealth initiative (Yes/No)? 1b. “Is the CFIR combined with other frameworks in assessing the telehealth initiative (Yes/No)?” and 1c. “Which CFIR domains (or constructs) were identified as influential in explain telehealth implementation effectiveness?” Each data item in turn, was directly aligned with the corresponding review questions (RQs 1a, 1b, and 1c) outlined earlier.

Data items relevant to RQ 2 (“*What are the descriptive characteristics of CFIR applications to telehealth implementation initiatives?*”) were as follows: 2a. Healthcare Domains of Interest; 2b. Targeted Diagnoses or Conditions; 2c. Targeted Patient Populations; 2d. Technology Areas; and 2e. Service Areas of Interest. Each data item in turn, was directly aligned with the corresponding review questions (RQs 2a, 2b, 2c, 2d, and 2e) outlined earlier.

### Process for synthesizing results

Data were summarized using counts, aggregates, and proportions for analysis based on the review questions. For example, data on article characteristics (e.g., article type and publication year) and data on review questions (e.g., CFIR domains found to influence telehealth implementation) were summarized for analysis and interpretation. This process in turn, helped to synthesize results and draw inferences related to the state of the science on CFIR applications to telehealth implementation initiatives.

## Results

### Selection of sources of evidence

**Fig. 1:**
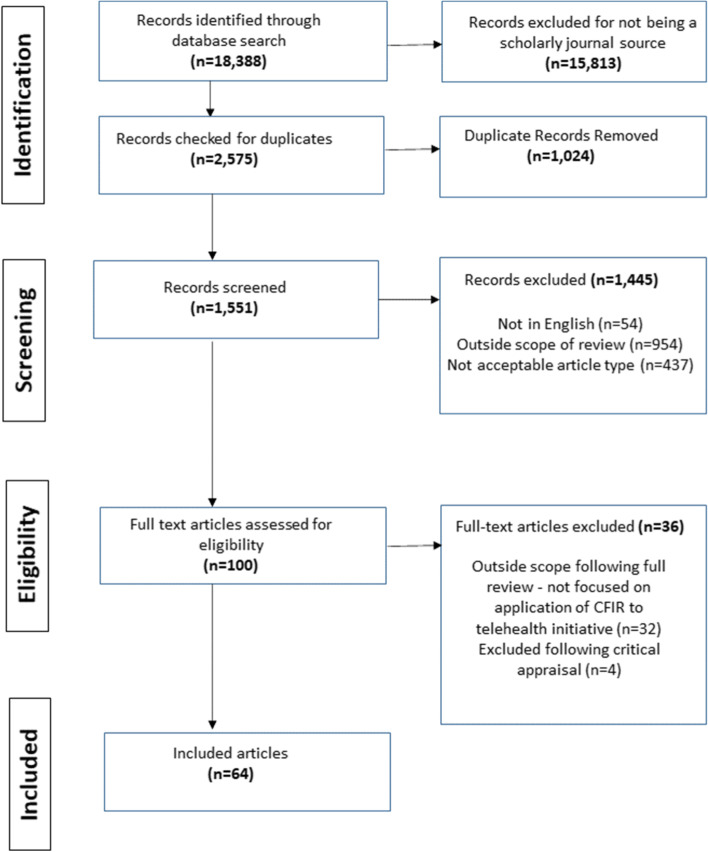
PRISMA Article Selection Flow Chart

The initial database search resulted in a total of 18,388 records. Of these, a total of 15,813 records were excluded for not being peer-reviewed journal articles. Among the remaining 2575 peer-reviewed journal articles, a total of 1024 duplicates were removed, and the remaining 1551 articles were screened for eligibility based on titles & abstracts. Of these, 54 articles were excluded for being wholly or partially in non-English language. An additional 954 articles were excluded for being outside the scope of the review; and an additional 437 articles were excluded for not being an acceptable article type (including 94 study protocols). The remaining 100 articles were retrieved in full text for assessing eligibility for inclusion. Following full review, 32 articles were excluded for being outside the scope of the study, and the remaining 68 articles were subjected to critical appraisal. Following critical appraisal, a total of 64 articles were identified for final inclusion in the scoping review [24–87]. The search results are summarized in full in a PRISMA chart in Fig. 1. The supplementary material includes a breakdown of the aggregate search results for each of the five databases (Additional file 6).

### Characteristics of sources of evidence

Table 1 outlines data items representing characteristics of included articles. The results are synthesized below, and full article citations for all individual sources of evidence are provided under References.

**Table 1:** Data charting on article characteristics

#	Article Name	Authors	Publication Year	Article Description	Article Type
1	Evaluation of multi-level barriers and facilitators in a large diabetic retinopathy screening program in federally qualified health centers: a qualitative study	Bastos de Carvalho A, Lee Ware S, Belcher T, Mehmeti F, Higgins EB, Sprang R, Williams C, Studts JL, Studts CR.	2021	This qualitative study aimed to identify and evaluate multi-level perceived determinants of Telemedicine Diabetic Retinopathy Screening in Federally Qualified Health Centers.	Qualitative or Mixed-Method
2	Factors associated with successful implementation of telehealth abortion in 4 United States clinical practice settings	Godfrey EM, Fiastro AE, Jacob-Files EA, Coeytaux FM, Wells ES, Ruben MR, Sanan SS, Bennett IM.	2021	This study identifies organizational factors that promoted successful implementation of telehealth and adoption of “no test” medication abortion protocols.	Qualitative or Mixed-Method
3	An Implementation Strategy to Expand Mobile Health Use in HIV Care Settings: Rapid Evaluation Study Using the Consolidated Framework for Implementation Research	Cohn WF, Canan CE, Knight S, Waldman AL, Dillingham R, Ingersoll K, Schexnayder J, Flickinger TE.	2021	This study conducted an interim analysis of the barriers and facilitators to PositiveLinks implementation at early adopting sites to guide optimization of the implementation strategy.	Qualitative or Mixed-Method
4	Staff Perceptions of Pre-implementation Barriers and Facilitators to a Mobile Health Antiretroviral Therapy Adherence Counseling Intervention in South Africa: Qualitative Study	McCreesh-Toselli S, Torline J, Gouse H, Robbins RN, Mellins CA, Remien RH, Rowe J, Peton N, Rabie S, Joska JA.	2021	This study explores staff’s perceived pre-implementation barriers and facilitators of an mHealth intervention developed as a structured app for ART readiness counseling, at a large Cape Town-based nonprofit HIV care organization.	Qualitative or Mixed-Method
5	Healthcare stakeholders’ perceptions and experiences of factors affecting the implementation of critical care telemedicine (CCT): qualitative evidence synthesis	Xyrichis A, Iliopoulou K, Mackintosh NJ, Bench S, Terblanche M, Philippou J, Sandall J.	2021	The aim was to identify, appraise and synthesize qualitative research evidence on healthcare stakeholders’ perceptions and experiences of factors affecting the implementation of Critical Care Telemedicine CCT.	Systematic Review
6	Barriers and Facilitators for Implementing Paediatric Telemedicine: Rapid Review of User Perspectives	Tully L, Case L, Arthurs N, Sorensen J, Marcin JP, O’Malley G.	2021	This study conducts a rapid mixed-methods evidence synthesis to identify barriers, facilitators, and documented stakeholder experiences of implementing pediatric telemedicine, to inform the pandemic response.	Systematic Review
7	What Drives Greater Assimilation of Telestroke in Emergency Departments?	Uscher-Pines L, Sousa J, Zachrison K, Guzik A, Schwamm L, Mehrotra A.	2020	Although many emergency departments (EDs) have tele-stroke capacity, it is unclear why some EDs consistently use tele-stroke and others do not. This study compared the characteristics and practices of EDs with robust and low assimilation of tele-stroke.	Qualitative or Mixed-Method
8	Implementing mHealth Interventions in a Resource-Constrained Setting: Case Study from Uganda	Meyer AJ, Armstrong-Hough M, Babirye D, Mark D, Turimumahoro P, Ayakaka I, Haberer JE, Katamba A, Davis JL.	2020	This study aimed to characterize the challenges encountered in implementing a complex mHealth intervention in Uganda.	Implementation effectiveness study
9	Implementing a Digital Tool to Support Shared Care Planning in Community-Based Mental Health Services: Qualitative Evaluation	Pithara C, Farr M, Sullivan SA, Edwards HB, Hall W, Gadd C, Walker J, Hebden N, Horwood J.	2020	The aim of this study was to examine mental health care providers’ views of and experiences with the CPT during the pilot implementation phase and identify factors influencing its implementation.	Qualitative or Mixed-Method
10	Increasing buprenorphine access for veterans with opioid use disorder in rural clinics using telemedicine.	Brunet N, Moore DT, Lendvai Wischik D, Mattocks KM, Rosen MI.	2020	This study describes barriers, facilitators and lessons learned while implementing a system to remotely prescribe buprenorphine to Veterans in rural settings.	Qualitative or Mixed-Method
11	Barriers and facilitators in implementing a pilot, pragmatic, telemedicine-delivered healthy lifestyle program for obesity management in a rural, academic obesity clinic.	Batsis JA, McClure AC, Weintraub AB, Sette D, Rotenberg S, Stevens CJ, Gilbert-Diamond D, Kotz DF, Bartels SJ, Cook SB, Rothstein RI.	2020	This study aimed to understand barriers and facilitators of implementing a telemedicine-delivered tertiary-care, rural academic weight-loss program for the management of obesity.	Qualitative or Mixed-Method
12	A novel in situ simulation framework for introduction of a new technology: the 3-Act-3-Debrief model.	Barker LT, Bond WF, Vincent AL, Cooley KL, McGarvey JS, Vozenilek JA, Powell ES.	2020	A simulation-based introduction to new technologies provides opportunity to intentionally address specific factors that influence adoption.	Qualitative or Mixed-Method
13	e-Consult implementation success: lessons from 5 county-based delivery systems	Knox M, Murphy EJ, Leslie T, Wick R, Tuot DS.	2020	This study evaluates organizational factors for e-consult implementation across 5 publicly financed, county-based health systems in California.	Qualitative or Mixed-Method
14	The current use of telehealth in ALS care and the barriers to and facilitators of implementation: a systematic review	Helleman J, Kruitwagen ET, van den Berg LH, Visser-Meily JMA, Beelen A.	2020	This study aimed to provide an overview of telehealth used in the care for patients with amyotrophic lateral sclerosis (ALS) and identify the barriers to and facilitators of its implementation.	Systematic Review
15	Barriers and Facilitators to the Implementation of a Mobile Insulin Titration Intervention for Patients with Uncontrolled Diabetes: A Qualitative Analysis	Rogers E, Aidasani SR, Friedes R, Hu L, Langford AT, Moloney DN, Orzeck-Byrnes N, Sevick MA, Levy N.	2019	This study aimed to conduct a qualitative evaluation assessing barriers to and the facilitators of the implementation of the Mobile Insulin Titration Intervention (MITI) program into usual care.	Qualitative or Mixed-Method
16	Factors for Supporting Primary Care Physician Engagement with Patient Apps for Type 2 Diabetes Self-Management That Link to Primary Care: Interview Study	Ayre J, Bonner C, Bramwell S, McClelland S, Jayaballa R, Maberly G, McCaffery K.	2019	This study aimed to explore PCP perspectives on proposed features for a self-management app for patients with diabetes that would link to primary care services.	Qualitative or Mixed-Method
17	Factors affecting implementation of digital health interventions for people with psychosis or bipolar disorder, and their family and friends: a systematic review	Aref-Adib G, McCloud T, Ross J, O’Hanlon P, Appleton V, Rowe S, Murray E, Johnson S, Lobban F.	2019	This review sought to identify factors affecting implementation of digital health interventions for people affected by psychosis or bipolar disorder.	Systematic Review
18	Identifying Implementation Science Characteristics for a Prenatal Oral Health eHealth Application	Vamos CA, Green SM, Griner S, Daley E, DeBate R, Jacobs T, Christiansen S.	2020	This study aimed to identify key implementation science characteristics to inform the development of an eHealth application (app) to assist providers in implementing the prenatal oral health guidelines during prenatal visits.	Qualitative or Mixed-Method
19	Applying the consolidated framework for implementation research to identify barriers affecting implementation of an online frailty tool into primary health care: a qualitative study	Warner G, Lawson B, Sampalli T, Burge F, Gibson R, Wood S.	2018	This study used the CFIR as an evaluation framework during the piloting of a novel web-based tool called the Frailty Portal, developed to aid in the screening, identification, and care planning of frail patients in community PHC.	Qualitative or Mixed-Method
20	Telestroke Adoption Among Community Hospitals in North Carolina: A Cross-Sectional Study	Shea CM, Tabriz AA, Turner K, North S, Reiter KL.	2018	This study identifies community and hospital characteristics associated with adoption of tele-stroke among acute care hospitals in North Carolina.	Secondary Data Analysis
21	Operationalizing mHealth to improve patient care: a qualitative implementation science evaluation of the WelTel texting intervention in Canada and Kenya	Bardosh KL, Murray M, Khaemba AM, Smillie K, Lester R.	2017	In this study, we explored how different contextual factors influenced the implementation, effectiveness, and potential for scale-up of WelTel, an easy-to-use and evidence-based mHealth intervention.	Qualitative or Mixed-Method
22	Implementation of internet-delivered cognitive behavior therapy within community mental health clinics: a process evaluation using the consolidated framework for implementation research	Hadjistavropoulos HD, Nugent MM, Dirkse D, Pugh N.	2017	This paper reports on a parallel process evaluation designed to understand facilitators and barriers impacting the uptake and implementation of ICBT.	Qualitative or Mixed-Method
23	Evaluation of the Veterans Health Administration’s Specialty Care Transformational Initiatives to Promote Patient-Centered Delivery of Specialty Care: A Mixed-Methods Approach	Williams KM, Kirsh S, Aron D, Au D, Helfrich C, Lambert-Kerzner A, Lowery J, Battaglia C, Graham GD, Doukas M, Jain R, Ho PM.	2017	The evaluation, guided by two implementation frameworks, provides formative (administrator/provider interviews and surveys) and summative data (quantitative data on patterns of use) about the initiatives to OSC.	Qualitative or Mixed-Method
24	Evaluation of a national telemedicine initiative in the Veterans Health Administration: Factors associated with successful implementation	Stevenson L, Ball S, Haverhals LM, Aron DC, Lowery J.	2018	The aim of this study was to provide guidance and support for the implementation and spread of SCAN-ECHO.	Qualitative or Mixed-Method
25	Factors that influence the implementation of e-health: a systematic review of systematic reviews	Ross J, Stevenson F, Lau R, Murray E.	2016	This review provides a re-analysis of a systematic review of the e-health implementation literature culminating in a set of accessible and usable recommendations for anyone involved or interested in the implementation of e-health.	Systematic Review
26	Qualitative analysis of programmatic initiatives to text patients with mobile devices in resource-limited health systems	Garg SK, Lyles CR, Ackerman S, Handley MA, Schillinger D, Gourley G, Aulakh V, Sarkar U.	2016	Formative evaluations of texting implementation experiences are limited. This study interviewed safety net health systems piloting texting initiatives to study facilitators and barriers to real-world implementation.	Qualitative or Mixed-Method
27	E-consult implementation: lessons learned using consolidated framework for implementation research	Haverhals LM, Sayre G, Helfrich CD, Battaglia C, Aron D, Stevenson LD, Kirsh S, Ho M, Lowery J.	2015	This study conducted an evaluation to understand variation in the use of the new e-consult mechanism and the causes of variable implementation, guided by the CFIR.	Qualitative or Mixed-Method
28	Factors That Influence the Use of Electronic Diaries in Health Care: Scoping Review	Daniëls, Naomi E M;Hochstenbach, Laura M J;Catherine van Zelst;van Bokhoven, Marloes A;Philippe A E G Delespaul;Anna J H M Beurskens	2021	The aim of this study was to map the existing empirical knowledge and gaps concerning factors that influence the use of electronic diaries, defined as repeated recording of psychosocial or physical data lasting at least 1 w using a smartphone or a computer, in health care.	Systematic Review
29	A Digital Patient-Provider Communication Intervention (InvolveMe): Qualitative Study on the Implementation Preparation Based on Identified Facilitators and Barriers	Seljelid, Berit;Varsi, Cecilie;Lise Solberg Nes;Øystese, Kristin Astrid;Børøsund, Elin	2021	The aim of this study is to prepare for the implementation of a digital patient-provider communication intervention in the daily workflow at two outpatient clinics by identifying potential determinants of implementation using the CFIR.	Qualitative or Mixed-Method
30	A Clinical Communication Tool (Loop) for Team-Based Care in Pediatric and Adult Care Settings: Hybrid Mixed Methods Implementation Study	Husain A, Cohen E, Dubrowski R, Jamieson T, Kurahashi AM, Lokuge B, Rapoport A, Saunders S, Stasiulis E, Stinson J, Subramaniam S, Wegier P, Barwick M.	2021	The objective of this study was to implement and evaluate Loop. The study reporting adheres to the Standards for Reporting Implementation Research.	Implementation effectiveness study
31	Informing Proactive integrated virtual healthcare resource use in primary care	Haun JN, Cotner BA, Melillo C, Panaite V, Messina W, Patel-Teague S, Zilka B.	2021	The objectives were to identify proactive integrated VHR use among primary care teams, best practices, and targeted implementation strategies to promote proactive integrated VHR use.	Qualitative or Mixed-Method
32	“At least someone thinks I’m doing well”: a real-world evaluation of the quit-smoking app StopCoach for lower socio-economic status smokers	Meijer E, Korst JS, Oosting KG, Heemskerk E, Hermsen S, Willemsen MC, van den Putte B, Chavannes NH, Brown J.	2021	This study evaluated “De StopCoach”, a mobile phone delivered eHealth intervention targeted at lower-SES smokers based on the evidence-based StopAdvisor, in a real-world setting in The Netherlands.	Qualitative or Mixed-Method
33	Telemedicine for Remote Surgical Guidance in Endoscopic Retrograde Cholangiopancreatography: Mixed Methods Study of Practitioner Attitudes	Aminoff H, Meijer S, Arnelo U, Frennert S.	2021	The objective was to gain a deeper understanding of ERCP practitioners’ attitudes toward teleguidance. These findings could inform the implementation process and future evaluations.	Qualitative or Mixed-Method
34	Implementations of Evidence-Based eHealth Interventions for Caregivers of People with Dementia in Municipality Contexts (Myinlife and Partner in Balance): Evaluation Study	Christie HL, Boots LMM, Tange HJ, Verhey FRJ, de Vugt ME.	2021	This study’s objectives were to evaluate the success of the implementation of Myinlife and Partner in Balance and investigate determinants of their successful implementation in the municipality context.	Comparative effectiveness study
35	Implementation outcomes of Humanwide: integrated precision health in team-based family practice primary care	Brown-Johnson CG, Safaeinili N, Baratta J, Palaniappan L, Mahoney M, Rosas LG, Winget M.	2021	This study assessed implementation to inform spread and scale, using mixed methods of semi-structured interviews with diverse stakeholders and chart reviews.	Qualitative or Mixed-Method
36	Exploring factors that affect the uptake and sustainability of videoconferencing for healthcare provision for older adults in care homes: a realist evaluation	Newbould L, Ariss S, Mountain G, Hawley MS.	2021	This study explored factors affecting the uptake and sustainability of videoconferencing in care homes, to establish what works for whom, in which circumstances and respects.	Qualitative or Mixed-Method
37	Empowering Implementation Teams with a Learning Health System Approach: Leveraging Data to Improve Quality of Care for Transient Ischemic Attack	Rattray NA, Damush TM, Miech EJ, Homoya B, Myers LJ, Penney LS, Ferguson J, Giacherio B, Kumar M, Bravata DM.	2020	To identify how a virtual “Hub” dashboard that provided performance data for patients with transient ischemic attack (TIA), supported QI activities among newly formed multidisciplinary clinical teams at six Department of Veterans Affairs (VA) medical centers.	Qualitative or Mixed-Method
38	Referral to digital parent training in primary care: Facilitators and barriers.	Fehrenbacher, Caitlin;Schoeny, Michael E.;Reed, Monique;Shattell, Mona;Breitenstein, Susan M.	2020	This study sought to understand facilitators and barriers to the implementation of a referral to ezParent, a self-administered, digital PT program, in four primary care clinics from the perspective of clinic personnel.	Qualitative or Mixed-Method
39	Testing Consultation Recordings in a Clinical Setting with the SecondEars Smartphone App: Mixed Methods Implementation Study	Hyatt A, Lipson-Smith R, Morkunas B, Krishnasamy M, Jefford M, Baxter K, Gough K, Murphy D, Drosdowsky A, Phipps-Nelson J, White F, White A, Serong L, McDonald G, Milne D.	2020	This study aimed to pilot the SecondEars app within an active clinical setting to identify factors necessary for optimal implementation.	Qualitative or Mixed-Method
40	Evaluating the feasibility of implementing a Telesleep pilot program using two-tiered external facilitation	Rattray NA, Khaw A, McGrath M, Damush TM, Miech EJ, Lenet A, Stahl S, Ferguson J, Myers J, Guenther D, Homoya BJ, Bravata DM.	2020	This study aimed to evaluate the feasibility of implementing a clinical program that delivers treatment for OSA through PAP remote monitoring using external facilitation as an implementation strategy.	Comparative effectiveness study
41	The development of a theory-based eHealth app prototype to promote oral health during prenatal care visits	Vamos CA, Griner SB, Kirchharr C, Green SM, DeBate R, Daley EM, Quinonez RB, Boggess KA, Jacobs T, Christiansen S.	2019	The purpose of this study was to develop and test the usability of an innovative, theory-driven, eHealth application (“app”) to facilitate prenatal providers’ implementation of oral health promotion during prenatal care visits.	Qualitative or Mixed-Method
42	The Phased Implementation of a National Telehealth Weight Management Program for Veterans: Mixed-Methods Program Evaluation	Goodrich DE, Lowery JC, Burns JA, Richardson CR.	2018	The primary aim was to compare outcomes for TeleMOVE with standard, facility-based MOVE weight-management services over the evaluation period, as demonstrated by average weight lost per patient. The secondary aim was to understand factors influencing TeleMOVE implementation variability across demonstration sites.	Comparative effectiveness study
43	A qualitative study of implementation and adaptations to Progressive Tinnitus Management (PTM) delivery	Tuepker A, Elnitsky C, Newell S, Zaugg T, Henry JA.	2018	This study was designed to address a gap in knowledge of PTM clinical implementation to date, with a focus on factors facilitating or hindering implementation in VHA audiology and mental health clinic contexts, and whether implementing sites had developed intervention adaptations.	Qualitative or Mixed-Method
44	Why the uptake of eRehabilitation programs in stroke care is so difficult-a focus group study in the Netherlands	Brouns B, Meesters JJL, Wentink MM, de Kloet AJ, Arwert HJ, Vliet Vlieland TPM, Boyce LW, van Bodegom-Vos L.	2018	The aim of this study was to explore which factors influence the uptake of eRehabilitation in stroke rehabilitation, among stroke patients, informal caregivers, and healthcare professionals.	Qualitative or Mixed-Method
45	Can we address depression in vision rehabilitation settings? Professionals’ perspectives on the barriers to integrating problem-solving treatment	Holloway E, Sturrock B, Lamoureux E, Keeffe J, Hegel M, Casten R, Mellor D, Rees G.	2018	This study aims to identify the perceived barriers and facilitators to staff-delivered telephone-based problem-solving treatment for primary care (PST-PC) offered as an integrated component of LVR.	Qualitative or Mixed-Method
46	Barriers and facilitators to implementation of VA home-based primary care on American Indian reservations: a qualitative multi-case study	Kramer BJ, Cote SD, Lee DI, Creekmur B, Saliba D.	2017	The purpose of this study is to describe barriers and facilitators to implementation in rural Native communities with the aim of informing planners and policymakers for future program expansions.	Qualitative or Mixed-Method
47	Using the Consolidated Framework for Implementation Research to Identify Barriers and Facilitators for the Implementation of an Internet-Based Patient-Provider Communication Service in Five Settings: A Qualitative Study	Varsi C, Ekstedt M, Gammon D, Ruland CM.	2015	This study sought to identify and compare barriers and facilitators influencing the implementation of an IPPC service in 5 hospital units using the CFIR.	Qualitative or Mixed-Method
48	Evaluation of the initial implementation of a nationwide diabetic retinopathy screening programme in primary care: a multimethod study	Khou V, Khan MA, Jiang IW, Katalinic P, Agar A, Zangerl B.	2021	This study examined the benefits and barriers of a nationwide diabetic retinopathy screening program, image interpretation pathways and assessed the characteristics of people who had their fundus photos graded by a tele-reading service which was available as a part of the program.	Qualitative or Mixed-Method
49	Barriers and facilitators to development and implementation of a rural primary health care intervention for dementia: a process evaluation	Morgan D, Kosteniuk J, O’Connell ME, Kirk A, Stewart NJ, Seitz D, Bayly M, Froehlich Chow A, Elliot V, Daku J, Hack T, Hoium F, Kennett-Russill D, Sauter K.	2019	Using a community-based participatory research approach, researchers collaborated with a rural PHC team to co-design and implement an evidence-based interdisciplinary rural PHC memory clinic in the Canadian province of Saskatchewan. This paper reports barriers and facilitators to developing, implementing, and sustaining the intervention.	Qualitative or Mixed-Method
50	Patient and provider acceptance of telecoaching in type 2 diabetes: a mixed-method study embedded in a randomised clinical trial	Odnoletkova I, Buysse H, Nobels F, Goderis G, Aertgeerts B, Annemans L, Ramaekers D.	2016	The aim of the study was to explore the perceptions of patients, nurses, and general practitioners (GPs) regarding tele-coaching in type 2 diabetes.	Qualitative or Mixed-Method
51	An Evaluation of Cardiology Virtual Care During the COVID-19 Pandemic	Sanderson KE, Spithoff KD, Corovic M, Langdon KM, Schwalm JD.	2021	A survey evaluation was conducted in the division of cardiology at a tertiary care academic center to assess barriers, facilitators, acceptability, and feasibility of virtual care during the COVID-19 pandemic.	Qualitative or Mixed-Method
52	Dissemination and implementation science in program evaluation: A telemental health clinical consultation case example	Arora PG, Connors EH, Blizzard A, Coble K, Gloff N, Pruitt D.	2016	This paper seeks to inform program evaluation efforts by outlining two D&I frameworks and describing their integration in program evaluation design.	Qualitative or Mixed-Method
53	Teledentistry System in Dental Health Public Services: A Mixed-Methods Intervention Study	Böhm da Costa C, da Silva Peralta F, Aurelio Maeyama M, Goulart Castro R, Lúcia Schaefer Ferreira de Mello A.	2021	To analyze the factors that affect the implementation of a Tele-dentistry system in dental health public services.	Qualitative or Mixed-Method
54	Barriers and Facilitators to the Implementation of Virtual Reality as a Pain Management Modality in Academic, Community, and Safety-Net Settings: Qualitative Analysis	Sarkar, Urmimala; Lee, Jane E; Nguyen, Kim H; Lisker, Sarah; Lyles, Courtney R.	2021	We conducted a qualitative, theory-informed implementation science study to assess the readiness for VR in safety-net settings.	Qualitative or Mixed-Method
55	Implementation, Adoption, and Perceptions of Telemental Health During the COVID-19 Pandemic: Systematic Review	Appleton R, Williams J, Vera San Juan N, Needle JJ, Schlief M, Jordan H, Sheridan Rains L, Goulding L, Badhan M, Roxburgh E, Barnett P, Spyridonidis S, Tomaskova M, Mo J, Harju-Seppänen J, Haime Z, Casetta C, Papamichail A, Lloyd-Evans B, Simpson A, Sevdalis N, Gaughran F, Johnson S.	2021	To investigate the adoption and impacts of tele-mental health approaches during the COVID-19 pandemic, and facilitators and barriers to optimal implementation.	Systematic Review
56	Establishing Clinical Swallowing Assessment Services via Telepractice: A Multisite Implementation Evaluation	Ward EC, Burns CL, Gray A, Baker L, Cowie B, Winter N, Rusch R, Saxon R, Barnes S, Turvey J.	2021	Using an implementation framework, strategies that supported implementation of CSE services via tele-practice within 18 regional/rural sites across five health services were examined.	Qualitative or Mixed-Method
57	Barriers and facilitators to implementing telehealth services during the COVID-19 pandemic: A qualitative analysis of interviews with cystic fibrosis care team members	Van Citters AD, Dieni O, Scalia P, Dowd C, Sabadosa KA, Fliege JD, Jain M, Miller RW, Ren CL.	2021	Our objective was to provide a qualitative exploration of facilitators and barriers to 1) implementing high-quality telehealth and 2) navigating reimbursement for telehealth services.	Qualitative or Mixed-Method
58	The efficacy, challenges, and facilitators of telemedicine in post-treatment cancer survivorship care: an overview of systematic reviews	J Chan, M Crichton, F Crawford-Williams, O A Agbejule, K Yu, N H Hart, F de Abreu Alves, F D Ashbury, L Eng, M Fitch, H Jain, M Jefford, D Klemanski, B Koczwara, K Loh, M Prasad, H.	2021	To inform guidance for the use of telemedicine in the post-COVID era, the aim of this overview of this systematic review was to evaluate the efficacy of, and survivor engagement in, telemedicine interventions in the post-treatment survivorship phase, and to consider implementation barriers and facilitators.	Systematic Review
59	Evaluating the Implementation of a Mobile Phone-Based Telemonitoring Program: Longitudinal Study Guided by the Consolidated Framework for Implementation Research	Ware P, Ross HJ, Cafazzo JA, Laporte A, Gordon K, Seto E.	2018	The objective of this study was to evaluate the implementation of a mobile phone-based telemonitoring program, which has been implemented as part of standard care in a specialty heart function clinic.	Qualitative or Mixed-Method
60	Adapting the Consolidated Framework for Implementation Research to Create Organizational Readiness and Implementation Tools for Project ECHO	Serhal E, Arena A, Sockalingam S, Mohri L, Crawford A.	2021	This study sought to (1) create a set of questions to assess organizational readiness and suitability of the ECHO model and (2) provide those who have determined ECHO is the correct model with a checklist to support successful implementation.	Qualitative or Mixed-Method
61	Considerations for the Implementation of a Telestroke Network: A Systematic Review	Tumma A, Berzou S, Jaques K, Shah D, Smith AC, Thomas EE.	2021	Given the prolific expansion of tele-stroke services since 2010, we conducted a systematic review to determine factors associated with successful establishment, management, and sustainability of a contemporary tele-stroke services.	Systematic Review
62	Using patient-reported outcome measures during the management of patients with end-stage kidney disease requiring treatment with hemodialysis (PROM-HD): a qualitative study	Anderson NE, McMullan C, Calvert M, Dutton M, Cockwell P, Aiyegbusi OL, Kyte D.	2021	This study investigates the use of electronic formats (ePROs) to maximize the potential of PRO use, through exploration of the experiences, views and perceptions of patients and healthcare professionals (HCPs) on implementation and use of PROs in hemodialysis settings.	Qualitative or Mixed-Method
63	Evaluation of the implementation process of the mobile health platform ‘WelTel’ in six sites in East Africa and Canada using the modified consolidated framework for implementation research (mCFIR)	El Joueidi S, Bardosh K, Musoke R, Tilahun B, Abo Moslim M, Gourlay K, MacMullin A, Cook VJ, Murray M, Mbaraga G, Nsanzimana S, Lester R.	2021	To identify the facilitators and barriers for implementing WelTel and to assess the application of the mCFIR tool in facilitating focus groups in different geographical and health settings.	Qualitative or Mixed-Method
64	Teleneurology Expansion in Response to the COVID-19 Outbreak at a Tertiary Health System in New York City	Kummer, BR; Sweetnam, C; Vickrey, BG; Naasan, G; Harvey, D; Gallagher, K	2021	To assess the implementation of tele-neurology (TN), including patient and clinician experiences, during the coronavirus respiratory disease 2019 (COVID-19) pandemic.	Qualitative or Mixed-Method

### Results of individual sources of evidence

Table 2 outlines data items representing results as they relate to review questions, including RQ1. The results are synthesized below.

**Table 2:** Data charting on review questions

#	RQ 1: What have we learned so far from applications of the CFIR to telehealth service implementation initiatives?			RQ 2: What are the descriptive characteristics of CFIR applications to telehealth service implementation initiatives?				
	***Includes a Summary Measure of Implementation Effectiveness of Telehealth Initiative (Yes/No)?***	***Which CFIR Domains serve to Explain Implementation Success or Failure of Telehealth Initiative?***	***Is CFIR Combined with Other Frameworks in Assessment of Telehealth Initiative (Yes/No)?***	***Healthcare Domain***	***Targeted Diagnoses or Conditions***	***Targeted Patient Populations***	***Technology Areas***	***Service Areas***
1	No	Inner Setting, Process	No	Primary Care	Diabetes Related (Retinopathic Screening)	Adults	Synchronous; Telemedicine for Diabetic Retinopathy Screening (TDRS); Interactive Audio/Video	Clinical Practice, Population Health, FQHCs
2	No	Inner Setting	No	Specialty Care	Maternal Health (Abortion)	Pregnant women	Synchronous or Asynchronous; Tele-Abortion Services	Clinical Practice
3	No	Inner Setting, Intervention Characteristics, Process	No	Specialty Care	Human Immunodeficiency Virus (HIV)	Adults	Asynchronous; mHealth; App; Self-care for people living with HIV	Clinical Practice, Population Health (Self-Management in Vulnerable Populations)
4	No	Inner Setting, Intervention Characteristics	No	Specialty Care	Human Immunodeficiency Virus (HIV)	Adults	Asynchronous; mHealth; App; ART therapy readiness counseling	Clinical Practice
5	No	Inner Setting, Individual Characteristics, Process	No	Acute & Intensive Care	Critical Care	Adults	Synchronous or Asynchronous; Critical Care Telemedicine	Clinical Practice
6	No	Inner Setting, Individual Characteristics, Process	No	Pediatrics	Pediatric Care (All Levels)	Children	Synchronous or Asynchronous; Tele-Stroke Care	Clinical Practice
7	No	Inner Setting, Individual Characteristics, Process	No	Emergency Care	Stroke	Seniors (Elderly)	Synchronous or Asynchronous; Pediatric Telemedicine	Clinical Practice
8	Yes	Inner Setting, Intervention Characteristics, Individual Characteristics	No	Specialty Care	Tuberculosis (TB)	Adults	Asynchronous; Remote Monitoring for TB Contact Investigation	Clinical Practice
9	No	Inner Setting, Intervention Characteristics, Individual Characteristics	No	Specialty Care	Mental Health/Psychiatry	Adults	Asynchronous; mHealth; Mobile Digital Care Pathway Tool for Tele-Mental Health	Clinical Practice, Population Health (Community-Based Mental Health Services)
10	No	Inner Setting, Individual Characteristics, Process	No	Specialty Care	Mental Health/Psychiatry (Substance Abuse)	Veterans	Synchronous or Asynchronous; Tele-Prescribing; Interactive Audio/Video	Clinical Practice, Medical Education
11	No	Inner Setting, Individual Characteristics, Process	Yes	Specialty Care	Lifestyle Health (Obesity Management)	Adults	Synchronous or Asynchronous; Telemedicine-Delivered Healthy Lifestyle Program for Obesity Management	Clinical Practice, Medical Education, Population Health (Reduction of Rural Health Disparities)
12	No	Inner Setting	No	Emergency Care	Emergency Management	Adults	Synchronous; eICU for Emergency Department	Clinical Practice, Medical Education, Population Health (Reduction of Rural Healthcare Access Disparities)
13	No	Inner Setting, Intervention Characteristics	No	Specialty Care	Specialty Care Referrals (eConsults)	All ages	Synchronous or Asynchronous; eConsults	Medical Education
14	No	Inner Setting, Process	No	Specialty Care	Mental Health/Psychiatry (Dementia)	Adults	Synchronous or Asynchronous; Telemedicine for ALS Care	Clinical Practice
15	No	Inner Setting, Intervention Characteristics, Individual Characteristics	No	Primary Care	Diabetes Related (Chronic Disease Management)	Adults	Asynchronous; mHealth; Messaging Device	Clinical Practice
16	No	Intervention Characteristics, Individual Characteristics, Process	No	Primary Care	Diabetes Related (Chronic Disease Management)	Adults	Asynchronous; mHealth; App; Mobile Apps	Clinical Practice
17	No	Inner Setting, Intervention Characteristics, Individual Characteristics	No	Specialty Care	Mental Health/Psychiatry (Psychosis)	Adults	Synchronous or Asynchronous; Digital Health Tools for People Affected by Psychosis or bipolar disorder	Clinical Practice
18	No	Inner Setting, Individual Characteristics, Outer Setting	No	Oral Health	Dentistry	Adults	Synchronous or Asynchronous; eHealth	Clinical Practice
19	No	Inner Setting, Intervention Characteristics, Individual Characteristics	No	Primary Care	Geriatrics (Frailty)	Seniors (Elderly)	Asynchronous; Web-Based Tool for Screening for Frailty	Clinical Practice
20	No	Inner Setting, Outer Setting	No	Emergency Care	Stroke	Seniors (Elderly)	Synchronous or Asynchronous; Tele-Stroke Care	Clinical Practice
21	No	Inner Setting, Intervention Characteristics, Individual Characteristics, Process	No	General (All)	Patient Engagement (Medication Adherence)	Adults	Asynchronous; SMS Texting for Medication Adherence	Clinical Practice
22	No	Inner Setting, Intervention Characteristics, Process	No	Specialty Care	Mental Health/Psychiatry	Adults	Synchronous or Asynchronous; Tele-Psychiatry	Clinical Practice, Population Health (Community-Based)
23	No	Inner Setting	No	Specialty Care	Specialty Care Referrals (eConsults)	Veterans	Synchronous or Asynchronous; eConsults	Clinical Practice, Medical Education, Population Health (Specialty Care Neighborhood)
24	No	Inner Setting, Intervention Characteristics	No	Specialty Care	Specialty Care Referrals (eConsults)	Veterans	Synchronous or Asynchronous; eConsults	Clinical Practice, Medical Education, Population Health (Specialty Care Neighborhood)
25	No	Inner Setting, Individual Characteristics, Outer Setting, Process	No	General (All)	eHealth (All Levels)	All ages	Synchronous or Asynchronous; eHealth	Clinical Practice
26	No	Outer Setting, Process	No	Primary Care	Patient Engagement (Medication Adherence)	Adults	Asynchronous; SMS Texting for Medication Adherence	Population Health (Safety Net & Health Equity)
27	No	Inner Setting, Intervention Characteristics	No	Specialty Care	Specialty Care Referrals (eConsults)	Veterans	Synchronous or Asynchronous; eConsults	Clinical Practice
28	No	Intervention Characteristics, Individual Characteristics, Process	No	Specialty Care	Mental Health/Psychiatry	Adults	Asynchronous; mHealth; Remote Monitoring; Electronic Diaries	Clinical Practice
29	No	Inner Setting, Intervention Characteristics, Process	No	Specialty Care	Mental Health/Psychiatry	Adults	Asynchronous; mHealth; Remote Monitoring; Electronic Diaries	Clinical Practice
30	Yes	Intervention Characteristics, Individual Characteristics, Outer Setting, Process	No	Acute & Intensive Care	Patient-Provider Communication	Adults	Synchronous or Asynchronous; Team-Based Communication, Patients and Caregivers	Clinical Practice
31	No	Inner Setting, Intervention Characteristics	No	Primary Care	Provider-to-Provider Communication (Team-Based Care)	Adults	Asynchronous; Team-Based Care; Virtual Digital Health	Clinical Practice
32	No	Intervention Characteristics, Process	No	Specialty Care	Lifestyle Health (Smoking Cessation)	Adults	Asynchronous; mHealth; App; Smoking Cessation	Clinical Practice
33	No	Inner Setting, Individual Characteristics, Process	No	Specialty Care	Surgery (Surgical Guidance)	Adults	Synchronous or Asynchronous; Digital Health; Surgical Guidance	Clinical Practice, Medical Education
34	YES	Inner Setting, Individual Characteristics	No	Specialty Care	Mental Health/Psychiatry	Seniors (Elderly)	Synchronous or Asynchronous; eHealth	Clinical Practice
35	No	Inner Setting, Individual Characteristics, Process	No	Primary Care	Precision Health	Adults	Synchronous or Asynchronous; Precision Health; Interactive and Remote	Clinical Practice
36	No	Inner Setting, Individual Characteristics	No	Specialty Care	Geriatrics	Seniors (Elderly)	Synchronous; Geriatric Care; Interactive Audio/Video	Clinical Practice
37	No	Inner Setting, Process	No	Specialty Care	Cardiology	Veterans	Asynchronous; Team-Based Care; Web-based Virtual Hub for QI	Clinical Practice
38	No	Inner Setting, Intervention Characteristics	No	Pediatrics	Parent Training (School-Based)	Adults	Asynchronous; Digital Health; Referral to Digital Parent Training in Primary Care	Clinical Practice
39	No	Inner Setting, Outer Setting	No	Specialty Care	Oncology	Adults	Asynchronous; mHealth; App; Patients to Record Doctor Consultations	Clinical Practice
40	Yes	Inner Setting, Intervention Characteristics	No	Specialty Care	Lifestyle Health (Sleep Medicine)	Adults	Asynchronous; Remote Monitoring of Sleep	Clinical Practice
41	No	Inner Setting, Intervention Characteristics	No	Oral Health	Dentistry	Adults	Synchronous or Asynchronous; eHealth; App for Oral Health during Prenatal Visits	Clinical Practice
42	Yes	Inner Setting	No	Specialty Care	Lifestyle Health (Obesity Management)	Veterans	Asynchronous; Remote Messaging Device for Weight Loss	Clinical Practice
43	No	Inner Setting, Intervention Characteristics, Process	No	Specialty Care	Mental Health/Psychiatry (Audiology)	Veterans	Synchronous or Asynchronous; Interactive and Remote Telemedicine for Audiology	Clinical Practice
44	No	Inner Setting, Intervention Characteristics	No	Post-Acute Care	Stroke Rehabilitation	Adults	Synchronous or Asynchronous; eHealth; Rehabilitation	Clinical Practice
45	No	Inner setting, Process	No	Specialty Care	Mental Health/Psychiatry	Adults	Synchronous or Asynchronous; Interactive Audio; Voice-Only Telehealth	Clinical Practice
46	No	Inner Setting, Intervention Characteristics, Process	No	Primary Care	Home-Based Primary Care	Veterans	Synchronous or Asynchronous; Home-Based Primary Care	Clinical Practice
47	No	Inner Setting, Individual Characteristics	No	Acute & Intensive Care	Patient-Provider Communication	Adults	Asynchronous; Digital Health; Internet-Based Patient-Provider Communication	Clinical Practice
48	No	Inner Setting, Process	No	Primary Care	Diabetes Related (Retinopathy Screening)	Adults	Synchronous or Asynchronous; Digital Health	Clinical Practice
49	No	Inner Setting, Intervention Characteristics, Process	No	Specialty Care	Mental Health/Psychiatry (Dementia)	Adults	Synchronous; Interactive Audio/Video	Clinical Practice
50	No	Inner Setting, Intervention Characteristics, Process	No	Primary Care	Diabetes Related (Chronic Disease Management)	Adults	Synchronous or Asynchronous; Tele-Coaching for Diabetes; Interactive Audio/Video	Clinical Practice
51	No	Inner Setting, Intervention Characteristics, Process	No	Specialty Care	Cardiology	Adults	Asynchronous; Virtual Care During Pandemic	Clinical Practice
52	No	Inner Setting	No	Specialty Care	Mental Health/Psychiatry	Youth	Synchronous or Asynchronous; Telemedicine in Dentistry	Program Evaluation
53	No	Inner Setting	No	Oral Health	Dentistry	Adults	Synchronous or Asynchronous; Tele-Psychiatry for Youth	Clinical Practice
54	No	Inner Setting, Intervention Characteristics,	No	Specialty care	Pain Management in Safety Net Settings	Adults	Asynchronous; Virtual Reality	Clinical practice
55	No	Inner Setting, Intervention Characteristics, Individual Characteristics, Outer Setting, Process	No	Specialty care	Mental Health/Psychiatry	Adults	Synchronous or Asynchronous; Telemental Health	Clinical practice
56	No	Inner Setting	No	Specialty care	Speech and Language Pathology	Adults	Synchronous or Asynchronous; Telehealth for Cystic Fibrosis	Clinical practice
57	No	Inner Setting, Intervention Characteristics, Individual Characteristics, Outer Setting, Process	No	Specialty care	Cystic Fibrosis	All ages	Synchronous or Asynchronous; Clinical Swallowing Assessment Services	Clinical practice
58	No	Intervention Characteristics, Individual Characteristics	No	Specialty care	Oncology (Cancer-Survivorship Care)	Adults	Synchronous or Asynchronous; cancer survivorship	Clinical practice
59	No	Inner Setting, Individual Characteristics, Process	No	Specialty care	Heart Failure	Adults	Asynchronous; mHealth	Clinical practice
60	No	Inner Setting, Intervention Characteristics, Outer Setting	No	Specialty care	Complex disease management	Veterans	Synchronous or Asynchronous; Project ECHO	Clinical practice, medical education,
61	No	Inner Setting, Individual Characteristics, Process,	No	Specialty care	Stroke	Adults	Synchronous or Asynchronous; Kidney disease	Clinical practice
62	No	Inner Setting, Process	No	Specialty care	Kidney Disease	Adults	Asynchronous; mHealth	Clinical practice
63	No	Inner Setting, Individual Characteristics	No	Specialty care	Human Immunodeficiency Virus (HIV)	Adults	Asynchronous; ePROs for kidney disease	Clinical practice
64	No	Inner Setting, Individual Characteristics, Outer Setting, Process	No	Specialty care	Neurology	Adults	Asynchronous; Tele-Neurology	Clinical practice

### Synthesis of results

The data presented in Tables 1 and 2, provide a foundation for synthesizing the results of this review with respect to article characteristics and both the review questions (RQ1 and RQ2).

### Results based on key article characteristics

To begin with*,* Table 3 Part A provides a summary breakdown of articles reviewed by publication year, while Table 3 Part B provides a summary breakdown by article type. Table 3 Part A shows that a majority 64% of included articles were published in 2020 or later, with 20% published in 2020 and 44% published in 2021. This suggests that CFIR applications to telehealth have gained momentum during the pandemic period. Complementarily, the database search found several protocols for implementation-effectiveness studies on the topic of interest (published during/after 2020), which could not be included in this review. The latter in turn, serves to not only reinforce the gleaning that CFIR applications to telehealth initiatives received a boost during the pandemic, but also, that the science of telehealth implementation informed by the CFIR, has potential for significant advancement in the coming years, as study protocols materialize into completed and published studies.

**Table 3:** Summary of key article characteristics

	***#***	***%***
**Part A: Publication Year**		
2015	2	3%
2016	4	6%
2017	4	6%
2018	8	13%
2019	5	8%
2020	13	20%
2021	28	44%
**Total**	**64**	
**Part B: Article Type**		
Qualitative or Mixed-Method	49	77%
Systematic Review	9	14%
Implementation Effectiveness Study	2	3%
Comparative Effectiveness Study	3	5%
Other	1	2%
**Total**	**64**	

Table 3 Part B indicates that 85% (54) were research articles, while 14% (9) were review papers. The majority (77%) of all included articles (and 91% of research articles), were qualitative or mixed-method studies seeking to identify barriers or facilitators to telehealth service implementation informed by CFIR domains or constructs. In other words, most studies eligible for inclusion in this review, were focused on a qualitative or mixed-method assessment of barriers and facilitators to effective implementation of telehealth initiatives, using the CFIR. These studies in turn, were based on data collected from key informants involved in the implementation process, through interviews, focus groups, construct-based surveys, observation, content, or archival analysis and/or other mixed-method analytic techniques.

Among original research papers, examples of qualitative or mixed-method studies included the following: One study sought to evaluate perceived determinants of Telemedicine Diabetic Retinopathy Screening (TDRS) in Federally Qualified Health Centers, [24] through semi-structured interviews with key informants (administrators, clinicians, staff) involved in TDRS. Another study sought to identify organizational factors promoting successful implementation of telehealth and adoption of “no test” medication abortion protocols through semi-structured interviews with providers during the COVID-19 pandemic [25].

Among remaining original research papers, 3% (2) were implementation-effectiveness hybrid studies [31, 53]; 5% (3) were comparative effectiveness studies, [57, 63, 65] while only 2% (1) fell in the “other” category [43]. Implementation-effectiveness hybrid studies involved the concurrent evaluation of implementation and intervention effectiveness, and often included a qualitative or mixed-method design component for assessing implementation effectiveness. For example, one implementation-effectiveness study sought to implement and evaluate a complex mHealth intervention in Uganda [31]. Another sought to implement and evaluate a clinical communication tool (known as the Loop) [53] for team-based care in pediatric and adult care settings. Comparative effectiveness studies were clinical trials that sought to assess either the effectiveness of telehealth over usual care, or the comparative effectiveness of two different telehealth initiatives, or one initiative in different contexts. Like implementation-effectiveness hybrid studies, comparative effectiveness studies were often preceded or followed by a qualitative or mixed-method assessment of implementation effectiveness. For example, one comparative effectiveness study sought to compare outcomes for TeleMOVE with standard, facility-based MOVE weight-management services, while also examining factors influencing TeleMOVE implementation across demonstration sites [65]. The one paper that fell into the ‘other’ category, was a secondary data analysis study that sought to identify community and hospital characteristics associated with adoption of tele-stroke among acute care hospitals in the state of North Carolina, United States [43].

Examples of included review papers were as follows: one review paper sought to identify, appraise, and synthesize qualitative research evidence on healthcare stakeholders’ perceptions of factors affecting the implementation of Critical Care Telemedicine [28]. Another review sought to conduct a rapid mixed-methods evidence synthesis to identify barriers, facilitators, and stakeholder experiences of implementing pediatric telemedicine, to inform the pandemic response [29].

### Results based on review questions

With respect to results corresponding to the review questions, Table 4 Part A summarizes results pertaining to RQ1a (i.e., *if the study included an outcome measure of intervention or implementation effectiveness of the telehealth initiative*); Table 4 Part B summarizes results related to RQ1b (i.e., *if the study sought to supplement CFIR with another framework*); and Table 4 Parts C & D summarize results related to RQ1c (i.e., *which CFIR domains or constructs were identified to have influence over implementation effectiveness*).

**Table 4:** Summary of results on review question 1

	***#***	***%***
**Part A: Outcome Measure(s) Included (*Yes/No*)**		
Yes	5	8%
No	59	92%
**Total**	**64**	
**Part B: Supplemental Framework to CFIR Used (*Yes/No*)**		
Yes	1	2%
No	63	98%
**Total**	**64**	
**Part C: CFIR Domains of Influence (*Non-Exclusive Listing*)**		
Inner Setting by itself or among other domains	58	91%
Intervention Characteristics among other domains	32	50%
Individual Characteristics among other domains	28	44%
Outer Setting among other domains	9	14%
Process among other domains	33	52%
**Total**	**64**	
**Part D: CFIR Domains of Influence (*Exclusive Listing*)**		
Inner Setting Only	7	11%
Inner Setting, Individual Characteristics Only	4	6%
Inner Setting, Individual Characteristics, Outer Setting Only	1	2%
Inner Setting, Intervention Characteristics, Outer Setting Only	1	2%
Inner Setting, Individual Characteristics, Outer Setting, Process Only	2	3%
Inner Setting, Individual Characteristics, Process Only	9	14%
Inner Setting, Intervention Characteristics Only	10	16%
Inner Setting, Intervention Characteristics, Individual Characteristics, Process Only	1	2%
Inner Setting, Intervention Characteristics, Individual Characteristics, Outer Setting, Process Only	2	3%
Inner Setting, Intervention Characteristics, Individual Characteristics Only	5	8%
Inner Setting, Intervention Characteristics, Process Only	8	13%
Inner Setting, Outer Setting Only	2	3%
Inner Setting, Process Only	6	9%
Intervention Characteristics, Individual Characteristics Only	1	2%
Intervention Characteristics, Individual Characteristics, Outer Setting, Process Only	1	2%
Intervention Characteristics, Individual Characteristics, Process Only	2	3%
Intervention Characteristics, Process Only	1	2%
Outer Setting, Process Only	1	2%
**Total**	**64**	

Table 4 Part A shows that a very small proportion of articles 8% (5) included an outcome measure of intervention or implementation effectiveness, while the vast majority 92% did not. This ties in with the findings related to article type, i.e., that most included articles were qualitative studies seeking to examine barriers or facilitators to successful implementation through interactions (e.g., interviews) with key informants among high or low (sometimes, both high and low) implementers. In other words, these studies were not designed to provide outcome measure(s) of success; instead, study sites were selected based on prior observation of implementation success (e.g., usage rates) and were approached post-implementation for the qualitative assessment of barriers/facilitators for the purpose of gaining insight into strategies for pre-implementation readiness assessment in similar contexts. Like the qualitative studies, none of the review articles included outcome measures of intervention or implementation success.

Only the handful of implementation-effectiveness and comparative-effectiveness studies (reported earlier), included outcome measures. For example, one implementation-effectiveness study that sought to compare outcomes for TeleMOVE with standard, facility-based MOVE weight-management services over the evaluation period utilized both the number of patients enrolled per site and the program’s clinical effectiveness (as demonstrated by average weight lost per patient), as key outcome measures [65]. Concurrently, the study sought to understand factors influencing TeleMOVE implementation across demonstration sites. Another comparative effectiveness study that sought to evaluate the success of two health interventions for caregivers of people with dementia (Myinlife and Partner in Balance), [57] used a variety of outcome measures, including eHealth use data, coach evaluation questionnaires, and information on implementation determinants. Although studies incorporating outcome measures of telehealth implementation success were more the exception than the rule in this scoping review, it would be relevant to note that the database search found a growing number of protocols for implementation-effectiveness studies published since 2020 (36 protocols in 2020, and 58 in 2021, for a total of 94 protocols), which suggests that the science of CFIR applications to telehealth initiatives may be poised to generate additional outcome measures of intervention- or implementation-effectiveness in the near future.

Similarly, Table 4 Part B shows that only one article (2%) sought to supplement CFIR with other frameworks (e.g., the RE-AIM framework) to concurrently assess both implementation success and scalability, in the context of a telemedicine-delivered healthy lifestyle program for obesity management in a rural, academic obesity clinic [34]. The scarce use of additional frameworks to supplement CFIR in the context of telehealth initiatives, is unlike other healthcare implementation areas involving CFIR applications, like evidence-based practice implementation or health IT (Electronic Health Record) implementation, where CFIR has been more frequently supplemented with RE-AIM and other frameworks [19, 20].

Table 4 Part C summarizes the CFIR domains found to be of influence in telehealth implementation. As indicated in the table, most articles reviewed 58 (91%) reported the Inner Setting domain of CFIR to be significant in influencing telehealth implementation success, either by itself or alongside other CFIR domains. Only 6 (9%) of the articles reviewed did not report any findings related to Inner Setting. On the other hand, Inner Setting was found to be the sole domain of influence in 7 (11%) of the articles reviewed. Process, Intervention Characteristics, and Individual Characteristics domains, each followed the Inner Setting domain in being identified as influential predictors of telehealth implementation in 33(52%), 32(50%), and 28 (44%) of studies reviewed, respectively. By comparison, Outer Setting received the fewest mentions, with only 9 (14%) of articles reviewed identifying this domain to be of importance in influencing telehealth implementation success.

Given the predominance of Inner Setting in influencing telehealth implementation, it would be relevant to note the domains that appeared most frequently alongside Inner Setting. The Process domain appeared most frequently alongside the Inner Setting domain, with 28 mentions, while the Intervention Characteristics domain was a close second with 27 mentions alongside the Inner Setting domain, while Individual Characteristics received 24 mentions alongside Inner Setting. As shown in Table 4 Part D, the most frequent exclusive domain combinations were “Inner Setting and Intervention Characteristics,” which were found to be influential predictors of telehealth implementation success in 10 (16%) of the 64 studies, and “Inner Setting, Intervention Characteristics, and Process,” which were found to be influential in 9 (14%) of the 64 studies.

To elaborate, one study on determinants of Telemedicine Diabetic Retinopathy Screening in Federally Qualified Health Centers, found that four constructs, two related to Inner Setting (Leadership Engagement, and Goals and Feedback) and two related to Process (Engaging and Champion) were perceived as strongly distinguishing high from low implementation effectiveness [24]. Another study emphasized the relevance of Inner Setting as a facilitator, and Individual Characteristics as a barrier. This study compared the characteristics of EDs with robust and low implementation of tele-stroke and found the CFIR domain of Inner Setting to be of strong relevance to robust implementation [30]. In EDs with robust assimilation, tele-stroke programs had the support of leadership, tele-stroke use and outcomes were measured, and stakeholders received regular feedback about their tele-stroke use. By comparison, in EDs with low implementation, ED physicians felt that tele-stroke had little value beyond a telephone consult and tele-stroke was perceived to increase complexity, indicating that Individual Characteristics of implementers, served as a barrier to tele-stroke implementation. Another study seeking to identify barriers affecting implementation of an online frailty tool in primary care, [42] helped to understand the crucial role of Intervention Characteristics, specifically the potential for the intervention to be integrated into the workflow (i.e.,  intervention adaptability), in influencing implementation success.

One comparative-effectiveness study on telehealth over usual care, sought to understand barriers and facilitators to implementing a national telehealth weight management program (TeleMOVE) for Veterans [65]. Eleven sites reported high program complexity because TeleMOVE required more staff time per participant than MOVE! due to logistical and technical assistance issues related to the devices, indicating that Intervention Characteristics served as a barrier. High-uptake sites overcame implementation challenges by leveraging communication networks with stakeholders, setting programmatic goals, monitoring feedback of results, and taking time to foster incremental delivery improvements, all of which indicated how the Inner Setting could serve as a facilitator to implementation. On the other hand, low-uptake sites reported less leadership support and less communication among stakeholders, highlighting how the Inner Setting could serve as a barrier to implementation.

Next, Table 5 serves to summarize results pertaining to RQ2 (“*What are the descriptive characteristics of CFIR applications to telehealth initiatives?*”). Based on the sub-questions, Table 5 provides a summary breakdown of included articles by ‘healthcare domains of interest’ (Part A), ‘targeted diagnoses or conditions’ (Part B), ‘targeted populations’ (Part C), ‘technology areas of interest’ (Part D), and ‘service areas of interest’ (Part E).

**Table 5:** Summary of results on review question 2

	***#***	***%***
**Part A: Healthcare Domains of Interest**		
Specialty Care	40	63%
Primary Care	10	16%
Acute & Intensive Care	3	5%
Emergency Care	3	5%
Post-Acute Care	1	2%
Oral Health	3	5%
Pediatrics	2	3%
General (All)	2	3%
**Total**	**64**	
**Part B: Targeted Diagnoses and Conditions**		
Mental Health/Psychiatry	13	20%
Cardiology	2	3%
Critical Care	1	2%
Dentistry	3	5%
Complex Disease Management	1	2%
Diabetes Related (Chronic Disease Management)	5	8%
eHealth (All Levels)	1	2%
Emergency Management	1	2%
Geriatrics	2	3%
Home-Based Primary Care	1	2%
Human Immunodeficiency Virus (HIV)	3	5%
Lifestyle Health (Obesity Management)	4	6%
Maternal Health (Abortion)	1	2%
Oncology	2	3%
Parent Training (School-Based)	1	2%
Patient Engagement (Medication Adherence)	2	3%
Patient-Provider Communication	2	3%
Pediatric Care (All Levels)	1	2%
Precision Health	1	2%
Provider-to-Provider Communication (Team-Based Care)	1	2%
Specialty Care Referrals (eConsults)	4	6%
Stroke	4	6%
Surgery (Surgical Guidance)	1	2%
Tuberculosis (TB)	1	2%
Pain Management in Safety Net Settings	1	2%
Speech and Language Pathology	1	2%
Cystic Fibrosis	1	2%
Heart Failure	1	2%
Kidney Disease	1	2%
Neurology	1	2%
**Total**	**64**	
**Part C: Targeted Populations**		
Adults	44	69%
Veterans	9	14%
Seniors (Elderly)	5	8%
All Ages	3	5%
Children	1	2%
Pregnant women	1	2%
Youth	1	2%
**Total**	**64**	
**Part D: Technology Areas of Interest**		
Asynchronous only	25	39%
Synchronous or Asynchronous	35	55%
Synchronous only	4	6%
**Total**	**64**	
**Part E: Service Areas of Interest**		
Clinical Practice Only	50	78%
Clinical Practice, Medical Education	3	5%
Clinical Practice, Medical Education, Population Health	4	6%
Clinical Practice, Population Health	4	6%
Program Evaluation	1	2%
Medical Education	1	2%
Population Health (Safety Net & Health Equity)	1	2%
**Total**	**64**	

Table 5 Part A indicates that CFIR applications to telehealth initiatives have largely focused on the Specialty Care domain at 63% (40), followed by Primary Care at 16% (10), Emergency Care at 5% (3), Acute & Intensive Care at 5% (3), and Oral Health, also at 5% (3), followed by other domains. Table 5 Part B indicates that targeted diagnoses/conditions in Specialty Care consist of tele-psychiatry/mental health (including care for dementia, psychosis, audiology, and substance abuse), specialty care referrals (eConsults), tele-cardiology, telemedicine for infectious diseases (HIV, Tuberculosis), lifestyle health (including sleep medicine, smoking cessation, obesity management) geriatrics, maternal health, oncology, ophthalmology, and surgery. Targeted areas in the Primary Care domain, include diabetes (chronic disease) management, diabetic retinopathy screening, frailty screening, home-based primary care, precision health, and provider-to-provider communication (team-based care). Targeted areas in Emergency Care include stroke care and emergency management. Targeted areas in Acute & Intensive Care include patient-provider communication and Critical Care Telemedicine. Pediatrics included general pediatrics and school-based parent training, while the General domain included patient engagement and eConsults across various levels of care.

With respect to targeted populations, Table 5 Part C shows that CFIR applications to telehealth initiatives have largely focused on adults at 69% (including adults living with HIV, adults in rural areas, adults admitted to ICUs, and parents of school age children), followed by veterans at 14%, seniors at 8%, followed by children at 2%. Regarding technology areas of interest, Table 5 Part D shows that most CFIR Applications to telehealth initiatives have sought to leverage the benefits of both Synchronous and Asynchronous technologies at 55%, (e.g., Critical Care Telemedicine, eHealth for rehabilitation care, home-based primary care, pediatric telemedicine, precision health, tele-coaching for diabetes, lifestyle health promotion, including obesity management, tele-psychiatry for youth, and tele-stroke care). This was followed by a focus on Asynchronous-only technology at 31% (e.g., mHealth, remote monitoring, store-and-forward, virtual hub, and other asynchronous digital health initiatives, internet-based patient-provider communication, and SMS texting for medication adherence).

Regarding service areas of interest, Table 5 Part E shows that CFIR Applications to telehealth initiatives have focused mainly on the provision of healthcare delivery (i.e., clinical practice only) at 78%, with significantly lower proportions that have sought to use telehealth for 2) clinical practice and medical education (5%), 3) clinical practice and population health (6%), and 4) all three, i.e., clinical practice, medical education, and population health (6%).

## Discussion

### Summary of results

This review sought to characterize the scope of knowledge that has been gained thus far, from *applications of the CFIR to telehealth service implementation initiatives*. Following an extensive search for eligible articles in five major academic databases, a total of 64 peer-reviewed (original research or review) articles were reviewed. The review found that most (64% of) articles on the topic of interest have been published since 2020, and that a majority (77%) are qualitative or mixed-method studies seeking to identify barriers and facilitators to telehealth implementation (using CFIR), through interaction (e.g., interviews, focus groups, surveys) with key stakeholders involved in implementation. With respect to the scope of knowledge gained regarding success or failure of telehealth implementation initiatives, the review found a very small proportion of comparative or implementation-effectiveness studies (5%) that included outcome measure(s) of intervention or implementation effectiveness. Similarly, the review found that very few (2% of) studies sought to supplement the CFIR with other frameworks like the RE-AIM, to gain dual insight into implementation effectiveness and scalability/sustainability.

As discussed earlier, the CFIR’s potential as a comprehensive tool for informing factors influencing implementation-effectiveness has been demonstrated in other domains of health services innovation, including evidence-based practice (EBP) and Electronic Health Records (EHR) implementation [19, 20]. In this context, it would be relevant to acknowledge that research related to EBP and EHR implementation in the United States, has been directly proportional to the substantial attention these areas have received at a federal policy-level [6]. By comparison, telehealth has historically not been a federal health policy priority in the US. In the absence of policy-level support, telehealth adoption has historically been dictated by ad hoc initiatives undertaken at the individual provider or organizational level [1–3]. This changed during the COVID-19 pandemic, when telehealth received a much-needed boost from a combined surge in policy and provider-and-organizational-level attention, which in turn may help to explain the growth in research in this area during the pandemic, including the significant increase in study protocols for implementation-effectiveness trials on the topic of interest. These trends suggest that the science on the topic may be gravitating towards generating: 1) more outcome measures of telehealth implementation-effectiveness in the future, alongside 2) barriers and facilitators to implementation (based on CFIR domains) and 3) insights into the scalability and sustainability of telehealth initiatives using additional frameworks like RE-AIM, all of which could help to engender a more nuanced understanding of the determinants of telehealth implementation-effectiveness. These gleanings in turn suggest that a follow-up review on this topic within the next 5 years, may be a fruitful endeavor.

Regarding CFIR domains of influence, the Inner Setting domain was found to be significant in influencing telehealth implementation success in the vast majority (91%) of articles, either by itself, or alongside other domains. Several articles found the Inner Setting to be the sole domain to explain implementation success. On the other hand, none of the remaining four domains were found to be influential by themselves, which in turn serves to underscore the predominance of the Inner Setting domain in helping to explain telehealth implementation effectiveness. Inner Setting constructs that were found to be consistently important included, the availability of resources, goals & feedback, leadership engagement, readiness for implementation, and implementation climate.

The two domains that most frequently appeared alongside the Inner Setting domain were, Intervention Characteristics and Process. With respect to Intervention Characteristics, the construct of Adaptability (i.e., the ability to adapt the intervention to the prevailing context and more specifically, integrate the innovation it into the prevailing workflow), was consistently identified to be important. At a broader level, the intervention needs to align with the prevailing value system (among key stakeholders), for it to be successfully integrated with the workflow. The fact that Intervention Characteristics was often identified as a barrier or facilitator alongside Inner Setting, speaks to the importance of leadership engagement in ensuring intervention adaptability within the organizational context, to facilitate implementation success.

Along these lines it is noteworthy, that whenever the Process domain was found to be relevant, it was almost always accompanied by Inner Setting. Broadly, this helps to understand how implementation “process” goes together with the organizational “structure” (captured by the Inner Setting domain). This is helpful to understand when one considers that the absence of leadership engagement in an intervention, is likely to be accompanied by a lack of stakeholder engagement and championing, reflecting a failure of implementation Process. On the other hand, very few articles reviewed found the Outer Setting to (e.g., reimbursement policies for telehealth) to be of significance in explaining implementation effectiveness. This in turn suggests that permanent removal of policy and regulatory barriers to telehealth reimbursement by itself, may not suffice for ensuring implementation effectiveness. Instead, effective telehealth implementation requires healthcare providers to gain a more comprehensive understanding of implementation science dynamics, including the complex inter-relationships among the CFIR Inner Setting domains (and related constructs) and telehealth implementation effectiveness.

Regarding descriptive characteristics, the breakdown of articles by healthcare domain showed that majority of CFIR applications to telehealth initiatives have focused on the Specialty Care domain (63%), and the dominant targeted condition within Specialty Care was Mental Health (tele-psychiatry). There were fewer telehealth initiatives devoted to improving care coordination during transitions, patient-centered care, and team-based care. With respect to targeted populations, there has been greater focus on adults (69%) and veterans (14%), compared to children and youth (4%), and with respect to technology areas, most initiatives have relied on use of both synchronous and asynchronous technology (55%). Lastly, with respect to service area, the vast majority of CFIR applications to telehealth initiatives have focused on healthcare delivery (clinical practice), (78%) while a much lower proportion have concurrently also used telehealth for medical education and population health promotion.

### Implications for practice, policy, and future research

Findings by CFIR domain, suggest that simply focusing on issues impacting the Outer Setting, i.e., the removal of policy barriers including constraints associated with reimbursement and payment for telehealth services may not suffice in ensuring implementation-effectiveness. Instead, findings serve to underscore the predominant role of the Inner Setting domain (including leadership engagement, resources, measurement feedback, implementation readiness and implementation climate) in influencing implementation effectiveness. Findings also reveal that Process (stakeholder engagement and championing) related concerns are often accompanied by concerns associated with Inner Setting, implying that leadership engagement may be crucial in facilitating stakeholder engagement and cultivating champions for implementation.

Findings also point to the importance of interventional adaptability (integration into workflow) and individual characteristics, including perceptions and attitudes of individuals involved in influencing implementation success of telehealth initiatives. However, it would be relevant to note that like Process concerns, strategies for dealing with concerns associated with Intervention and Individual Characteristics often trace back to the Inner Setting, for example, one study found that leadership initiatives to strengthen relationships between stroke experts and ED providers helped to improve intervention adaptability, address resistant providers, and improve processes. From a practice perspective therefore, healthcare organizations need to understand these dynamics to be able to design and implement successful telehealth initiatives. From a policy perspective, both policy makers and advocacy groups, e.g., specialty society organizations such as the American Academy of Family Physicians, would be well advised to channel organized efforts and resources towards educating and training providers in implementation science dynamics to better prepare them for future success in telehealth implementation.

Regarding future research, the database search revealed several study protocols for telehealth implementation-effectiveness studies which could not be included in the review. Since these types of studies are frequently designed to provide outcome measures of implementation success, a follow-up review on the same topic in 5 years (to allow time for protocols to materialize into public studies), may help to better understand the determinants of implementation effectiveness, including the relationship between CFIR domains and success or failure of telehealth implementation. The trends in research growth on this topic may also align synergistically with the concurrent progress being made in conceptualizing outcomes for use with the CFIR, to provide an advanced understanding of the determinants of telehealth implementation-effectiveness [88]. In addition to a follow-up scoping review, a more mature evidence-base of outcome measures from completed implementation-effectiveness studies in the future, would have potential to provide a stronger foundation for systematic review efforts seeking to examine inter-relationships among a variety of CFIR domains & constructs within the telehealth implementation context, e.g., what is the relationship between implementation climate and effectiveness of telehealth implementation? Also, the findings related to descriptive characteristics of CFIR applications to telehealth implementation, by themselves, provide insight into gaps in the literature and potential avenues for future research growth, including the need for more CFIR applications to telehealth initiatives in primary care and acute care, as well as the need for telehealth initiatives that are focused on medical education and population health promotion, as opposed to a singular focus on clinical practice.

### Strengths and limitations

A key strength of this scoping review is that it helps to address a gap in the literature related to the scope of knowledge gained thus far from CFIR applications to telehealth implementation initiatives. Another strength was that the review was guided by evidence-based criteria for scoping reviews developed by the Joanna Briggs Institute and the internationally accepted guidelines for scoping reviews outlined in the PRISMA-ScR checklist. A clear rationale for use of scoping review (vs other types of review techniques) is provided at the outset, and the research questions of the scoping review are directly aligned with the review’s broader objective. Also, consistent with the rationale for a scoping review, the review involved a comprehensive database search of 5 major academic databases for individual sources of evidence (i.e., eligible journal articles) on the topic of interest.

One limitation of this review, however, is that other avenues for literature searches were not leveraged, e.g., 1) internet searches to examine the “gray literature” (industry publications, unpublished manuscripts, conference papers), 2) contact with authors to identify additional articles, and 3) review of reference lists of selected articles to identify additional articles. Additionally, the review found few implementation-effectiveness studies on the topic, which in turn limits insights gained into determinants of telehealth implementation success based on the CFIR. Nevertheless, the database search revealed an increasing number of protocols for implementation-effectiveness on the topic published in 2020 and 2021, suggesting that the science on this topic may be gravitating more towards generating additional outcome measures. This also implies that a follow-up review on the same topic within the next 5 years may be helpful in gaining advanced and nuanced insights on the relationship between CFIR domains and telehealth implementation-effectiveness.

## Conclusion

This scoping review reviewed 64 eligible articles to examine the scope of knowledge gained thus far from CFIR applications to telehealth implementation initiatives. The review found that most eligible articles were published in 2020 or later indicating that the science of CFIR applications to telehealth initiatives, has gained momentum during the pandemic. The review also found that most eligible articles were qualitative studies designed to examine barriers and facilitators to telehealth implementation through interaction (interviews, focus groups surveys etc.) with key stakeholders involved in implementation. By comparison, a very small proportion of articles were implementation- effectiveness studies that included outcome measures of intervention or implementation success. Similarly, very few articles used additional frameworks like the RE-AIM to supplement the CFIR to gain dual insights into implementation effectiveness and sustainability of telehealth initiatives. Regarding CFIR domains of influence, most included articles reported the Inner Setting domain to be of significance in influencing telehealth implementation effectiveness. By comparison, a very limited number of articles reported the Outer Setting to be of significance in influencing telehealth implementation. The review also found that most telehealth initiatives were undertaken in the specialty care domain, and that mental health/psychiatry was the most targeted condition. Most telehealth initiatives targeted adults (compared to children) and utilized both synchronous & asynchronous telemedicine technologies. The preponderance of articles also focused on utilizing telehealth for clinical practice, as opposed to medical education or population health.

A key takeaway is that to effectively design and implement telehealth initiatives, healthcare providers need to gain a thorough understanding of the inter-relationships between telehealth implementation effectiveness and CFIR domains, especially the CFIR Inner Setting domain and related constructs, including leadership engagement, resource availability, goals & feedback, and implementation climate. This suggests that policy advocacy groups and specialty societies, may need to place as much emphasis (if not more), on educating and training healthcare providers in their respective specialties on Inner Setting dynamics associated with telehealth implementation, in addition to advocating for better telehealth reimbursement policies, since the latter would only be relevant to addressing the Outer Setting domain of influence on telehealth implementation.

## Supplementary Information

**Additional file 1.**

**Additional file 2.**

**Additional file 3.**

**Additional file 4.**

**Additional file 5.**

**Additional file 6.**

## Data Availability

The data analyzed in this study is included in the supplementary material (Additional file 5 – Data Charting for RQ1 and RQ2).

## Abbreviations

ALS: Amyotrophic lateral sclerosis
CFIR: Consolidated Framework for Implementation Research
CCT: Critical Care Telemedicine
COVID-19: Coronavirus Disease 2019
ED: Emergency Department
e-Health: Electronic Healthcare
e-Rehabilitation: Electronic Rehabilitation Care
ERIC: Education Resources Information Center
FQHC: Federally Qualified Health Center
HIT: Health Information Technology
HIV: Human Immunodeficiency Virus
ICUs: Intensive Care Units
IEEE Explore: Engineering and Computer Science Digital Library
JBI: Joanne Briggs Institute
KDS: Khoja-Durrani-Scott Framework
mHealth: Mobile Health
PRISMA: Preferred Reporting Items for Systematic Reviews and Meta-Analyses
PRISMA-ScR: PRISMA extension for Scoping Reviews
RE-AIM: Reach, Effectiveness, Adoption, Implementation, Maintenance Framework
RQ: Review Question
SCIDIRECT: Science Direct
SMS: Short Message Service
TDRS: Telemedicine Diabetic Retinopathy Screening
UTAUT: The Unified Theory of Acceptance and Use of Technology
VA: Veterans Affairs
WoS: Web of Science
