# Supplementary material for: A scoping review of applications of the Consolidated Framework for Implementation Research (CFIR) to telehealth service implementation initiatives
Source: BMC Health Serv Res. 2022 Nov 30;22:1450. doi: 10.1186/s12913-022-08871-w (PMC9708146; doi:10.1186/s12913-022-08871-w)
Supplement: Supplementary file 1 — Additional file 1. [file 12913_2022_8871_MOESM1_ESM.docx]

**Review Protocol**

**A Scoping Review of Applications of the Consolidated Framework for Implementation Research (CFIR) to Telehealth Service Implementation Initiatives**

**Background**

The Consolidated Framework for Implementation Research (CFIR) introduced in 2009, has the potential to provide a holistic understanding of the determinants of implementation effectiveness in health service delivery. Although the CFIR has been increasingly used to understand factors influencing telehealth service implementation in recent years, no comprehensive review exists to-date on the scope of knowledge gained exclusively from applications of the CFIR to telehealth implementation initiatives. This scoping review sought to address this gap. The two broad review questions were: 1) What have we learned so far from CFIR applications to telehealth implementation initiatives? 2) What are the descriptive characteristics of CFIR applications to telehealth implementation initiatives?

**Review objective**

This paper undertakes a comprehensive review of the literature to characterize the scope of knowledge that has been gained thus far, from *applications of the Consolidated Framework for Implementation Research (CFIR) to telehealth service implementation initiatives*. The review objective is to “identify and synthesize the literature related to applications of CFIR to telehealth service implementation initiatives.” Correspondingly, this scoping review is expected to be directly relevant to healthcare providers and organizations looking to get started with telehealth and/or to design and implement telehealth services for effective and sustainable use.

**Review questions**

The specific review questions are outlined below.

1. **What have we learned so far from applications of the CFIR to telehealth service implementation initiatives?**
   1. What have we learned about the outcomes (success or failure) of telehealth service implementation initiatives?
   2. Has the CFIR been combined with other frameworks to enable assessment of both i) effectiveness and ii) scalability or sustainability of telehealth implementation?
   3. Which CFIR domains (or constructs) have been identified as most influential in explaining success or failure of telehealth implementation initiatives?
2. **What are the descriptive characteristics of CFIR applications to telehealth service implementation initiatives?**
   1. What healthcare domains (e.g., primary care, emergency care, post-acute care, mental health, oral health, etc.) have CFIR applications to telehealth initiatives focused on?
   2. What diagnoses or conditions (e.g., diabetes, stroke, cancer, depression, dementia etc.), have CFIR applications to telehealth initiatives focused on?
   3. What populations (e.g., children, adults, seniors, veterans, etc.), have CFIR applications to telehealth initiatives focused on?
   4. What technology areas (e.g., synchronous technologies such as interactive audio/video, or asynchronous technologies such as store-and-forward, remote monitoring, mHealth apps), have CFIR applications to telehealth focused on?
   5. What service areas (e.g., clinical practice/healthcare delivery, medical education, population health management, healthcare administration), have CFIR applications to telehealth initiatives focused on?

**Scoping review rationale**

According to Sucharew and Macaluso (2019), scoping reviews can be useful for answering broad questions, such as “What information has been presented on this topic in the literature?” which is fully consistent with what this review seeks to accomplish. To-date, there have been no comprehensive reviews of the literature to characterize the scope of knowledge that has been gained from *applications of the CFIR to telehealth service implementation initiatives*. This paper seeks to address this gap, and the review objective (and questions) in turn, are aligned with this purpose. Moreover, a scoping review is intended to provide an overview of the available research evidence without producing a summary answer to a discrete research question. The questions of this review lend themselves to a scoping review approach (versus other types of review), because they are broad in scope, and the review objective is to provide a descriptive account of available evidence on *applications of CFIR to telehealth service implementation initiatives*, as opposed to addressing a discrete research question (e.g., “what is the relationship between implementation climate and implementation success of telehealth service implementation initiatives?”) Also, a scoping review is performed when information on a topic has not been comprehensively reviewed, and the objective is to describe existing evidence, characteristics and knowledge gaps in a field or topic, which in turn is completely aligned with the objective of this review.

**Scoping review methodology**

Framework for the scoping review (PRISMA-ScR)

This paper conducts a scoping review of the literature to address the review questions. The review protocol was developed based on guidelines for scoping reviews provided by the Joanna Briggs Institute (JBI). The PRISMA-ScR criteria (for scoping reviews) were used to frame the review effort. The completed checklist is included in Appendix 2.

Information Sources

This scoping review sought to identify published original research papers (including quantitative, qualitative, and mixed-method studies) and review papers, to address the review questions. Since the CFIR was officially introduced in 2009, the following five major electronic academic databases were searched for coverage beginning 01.01.2010 through 12.31.21: 1) PUBMED, 2) SCIENCE DIRECT, 3) PROQUEST, 4) CINAHL, and 5) WEB OF SCIENCE (WoS). These databases were selected to ensure maximum coverage across medicine and social science domains. Additional searches were conducted on databases relevant to education and engineering domains, i.e., ERIC and IEEE Explore respectively. However, these searches produced negligible results on the topic of interest, and the latter two databases were excluded from information sources for this review. The search was conducted in March 2022.

Search Strategy

The following two sets of search terms were used to search all five databases for the period 01.01.2010 through 12.31.2021: **1)** (Telehealth OR Telemedicine) AND (Consolidated Framework for Implementation Research); and **2)** (Telehealth OR Telemedicine) AND (CFIR). It would be relevant to note that “Telemedicine” is a National Library of Medicine Medical Subject Heading (MeSH) term that includes the synonyms (entry terms) "mobile health," "mhealth" and "ehealth." The resulting total number of records from this initial search, for both sets of search terms combined, was 18,388 records (including 79 from PUBMED, 903 from SCIDIRECT, 17,318 from PROQUEST, 32 from CINAHL, and 56 from WoS). Both the combined and database totals included peer-reviewed (scholarly) journal articles, conference papers, working papers, wire feeds, reports, books, trade journals, dissertations, theses, magazines, and other sources. The next section describes the eligibility criteria that were applied to select articles for final inclusion in this scoping review.

Eligibility Criteria

This review considered original research articles (including clinical trials, quantitative, qualitative, and mixed-method studies) and review articles, that were published in peer-reviewed journals, in English language, and pertained to the scope of the review (i.e., ‘*the* *application of CFIR to telehealth implementation initiatives*’). Since the CFIR was officially introduced only in 2009, papers published between 01.01.10 and 12.31.21, were included for consideration. All forms of telehealth were considered, including telemedicine, digital health, eHealth and mHealth technologies. Research papers considered for inclusion were based on empirical data, including, but not limited to, data collected from clinical trials, surveys, observations, focus groups, and interviews. Among reviews, systematic and scoping reviews were considered for inclusion.

This scoping review excluded: 1) articles that were not published in peer-reviewed journals (e.g., conference papers, working papers, wire feeds, reports, books, trade journals, dissertations, theses, magazines, and other sources); 2) articles that were not pertinent to the review topic (e.g., papers that did not involve use CFIR or telehealth or both). It also excluded 3) articles that were neither original research nor reviews (e.g., study protocols, editorial articles, discussion papers, theoretical reflections, or any other type of article that did not include a methodology section). Additionally, this scoping review excluded 4) articles that did not meet critical appraisal criteria outlined by the Joanna Briggs Institute (JBI) for the relevant article type (e.g., qualitative studies, reviews, clinical trials, or cross-sectional studies).

Process for selecting sources of evidence

Following the search, all identified citations were collated and uploaded into a reference management system (Zotero 5.0) for initial screening. After removal of duplicates, article titles and abstracts were screened for potential inclusion, based on the eligibility criteria for the review. Articles identified for inclusion based on screening of titles & abstracts, were retrieved in full text for assessment based on the eligibility criteria. Reasons for article exclusion at each stage of the process were noted and have been reported in detail in the Results section. All articles that were selected based on eligibility criteria, were subjected to critical appraisal using the appropriate JBI checklist. Only articles that met the critical appraisal criteria were selected for final inclusion in the review. The templates used for article selection (i.e., eligibility and critical appraisal criteria), are included in the supplementary materials (Appendix 3). The results of the search are reported in full using a PRISMA flow chart.

Process for charting data items

All included articles were reviewed to retrieve two categories of data items, Category #1: data items for characterizing the articles (e.g., Article Name, Authors, Publication Year, Article Type); and Category #2: data items for capturing results based on the review questions (RQ1 and RQ2). Both data categories were retrieved from explicit information presented in the reviewed articles and charted in two separate spreadsheet templates included in Appendix 4. Correspondingly, Appendix 4 constitutes the raw dataset for the study. Together, the two data charting spreadhseets incorporated all the fields needed to capture the data items outlined above. No additional assumptions or simplifications needed to be made in the data charting process.

To elaborate, data items relevant to RQ 1 (“*What have we learned so far from applications of the CFIR to telehealth service implementation initiatives?*”) included: **1a.** “Does the article include an outcome measure of intervention or implementation effectiveness of the telehealth initiative (Yes/No)? **1b.** “Is the CFIR combined with other frameworks in assessing the telehealth initiative (Yes/No)?” and **1c**. “Which CFIR domains (or constructs) were identified as influential in explain telehealth implementation effectiveness?” Each data item in turn, was directly aligned with the corresponding review questions (RQs 1a, 1b, and 1c) outlined earlier.

Data items relevant to RQ 2 (“*What are the descriptive characteristics of CFIR applications to telehealth implementation initiatives?*”) were as follows: **2a.** Healthcare Domains of Interest; **2b.** Targeted Diagnoses or Conditions; **2c**. Targeted Patient Populations; **2d.** Technology Areas; and **2e**. Service Areas of Interest. Each data item in turn, was directly aligned with the corresponding review questions (RQs 2a, 2b, 2c, 2d, and 2e) outlined earlier.

Process for synthesizing results

Data were summarized using counts, aggregates, and proportions for analysis based on the review questions. For example, data on article characteristics (e.g., article type and publication year) and data on review questions (e.g., CFIR domains found to influence telehealth implementation) were summarized for analysis and interpretation. This process in turn, helped to synthesize results and draw inferences related to the state of the science on CFIR applications to telehealth implementation initiatives.
