# Supplementary material for: A scoping review of applications of the Consolidated Framework for Implementation Research (CFIR) to telehealth service implementation initiatives
Source: BMC Health Serv Res. 2022 Nov 30;22:1450. doi: 10.1186/s12913-022-08871-w (PMC9708146; doi:10.1186/s12913-022-08871-w)
Supplement: Supplementary file 3 — Additional file 3. [file 12913_2022_8871_MOESM3_ESM.docx]

**Full Electronic Search Strategy on PubMed**

<https://pubmed.ncbi.nlm.nih.gov/>

| **Search Terms** | **PubMed** |
| --- | --- |
| (Telehealth OR Telemedicine) AND (Consolidated Framework for Implementation Research) AND ("2010/01/01"[Date - Publication]: "2021/12/31"[Date - Publication]) | 52 |
| (Telehealth OR Telemedicine) AND (CFIR) AND ("2010/01/01"[Date - Publication]: "2021/12/31"[Date - Publication]) | 21 |
| **TOTAL** | 73 |

Following the electronic database search, specific article eligibility criteria and critical appraisal criteria (outlined on Page 10 of the manuscript and in Appendix 4), were applied to identify a final set of articles for inclusion in this scoping review.
